# The tyrosine phosphatase STEP is a developmental suppressor of synaptogenesis

**DOI:** 10.1073/pnas.2424788123

**Published:** 2026-06-10

**Authors:** Joel P. Pires, Diogo Tomé, Miranda Mele, Ana Caulino-Rocha, Elisa Corti, Ira Milosevic, Graça F. Baltazar, Ramiro D. Almeida

**Affiliations:** ^a^ Center for Neuroscience and Cell Biology, University of Coimbra, Coimbra 3004-504, Portugal; ^b^ Center for Innovative Biomedicine and Biotechnology, University of Coimbra, Coimbra 3004-504, Portugal; ^c^ Health Sciences Research Centre, University of Beira Interior, Covilhã 6200-506, Portugal; ^d^ RISE-Health, Department of Medical Sciences, Faculty of Health Sciences, University of Beira Interior, Covilhã 6200-506, Portugal; ^e^ Institute of Biomedicine, Department of Medical Sciences, University of Aveiro, Aveiro 3810-193, Portugal; ^f^ Multidisciplinary Institute of Aging, Center for Innovative Biomedicine and Biotechnology, University of Coimbra, Coimbra 3004-504, Portugal; ^g^ Faculty of Pharmacy, University of Coimbra, Coimbra 3000-548, Portugal; ^h^ Institute for Interdisciplinary Research, University of Coimbra, Coimbra 3030-789, Portugal; ^i^ Institute for Interdisciplinary Research, Doctoral Programme in Experimental Biology and Biomedicine, University of Coimbra, Coimbra 3030-789, Portugal; ^j^ Centre for Human Genetics, Nuffield Department of Medicine, University of Oxford, Oxford OX3-7BN, UK

**Keywords:** presynaptic differentiation, synapse formation, STEP phosphatase, fragile X syndrome, circuit-on-a-chip

## Abstract

Striatal-Enriched Protein Tyrosine Phosphatase (STEP) constrains synaptic potentiation by dephosphorylating postsynaptic substrates, but its presynaptic role has remained unclear. Here, we identify a previously unrecognized function of STEP in regulating axonal differentiation and synapse assembly. Genetic and pharmacological manipulation of STEP in vivo and in vitro show that STEP limits presynaptic maturation by restricting synaptic vesicle protein clustering along developing hippocampal axons. Using a reconstituted circuit-on-a-chip we show that loss of presynaptic STEP is sufficient to significantly increase the number of axodendritic synapses. Functional imaging further revealed that the increased synaptic puncta observed in STEP KO neurons actively undergo depolarization-evoked vesicle exocytosis, representing bona fide functional synapses. Multielectrode array recordings reveal that STEP deletion increases neuronal excitability, and network synchrony, hallmarks of enhanced presynaptic efficacy. Mechanistically, these effects reflect sustained phosphorylation of STEP promoting presynaptic assembly and release competence. Importantly, inhibiting STEP also rescues presynaptic differentiation defects in *Fmr1* KO neurons, implicating aberrant STEP signaling in Fragile X–associated synaptic pathology. Thus, STEP serves as a phosphatase gatekeeper that restrains presynaptic differentiation and neurotransmission, and its inhibition may offer a therapeutic strategy to correct synaptic deficits in Fragile X Syndrome.

## Significance

The balance between phosphorylation and dephosphorylation is essential for the signaling pathways that control synapse formation and maturation. Here, we identify Striatal-Enriched Protein Tyrosine Phosphatase (STEP) as a developmental brake that limits the formation and functional maturation of presynaptic terminals, the specialized structures that release neurotransmitters. Genetic and pharmacological suppression of STEP promotes synapse formation through a presynaptic-dependent mechanism, revealing a previously unrecognized role for STEP in shaping early circuit assembly. These findings identify STEP as a critical molecular regulator of synaptic development and a potential target for correcting synaptic dysfunction in Fragile X Syndrome.

Striatal-Enriched Protein Tyrosine Phosphatase (STEP) is a brain-specific phosphatase that plays an important role in regulating signaling pathways critical for neuronal development and synaptic plasticity. It is widely accepted that STEP opposes the development of synaptic strengthening by dephosphorylating and inactivating several target proteins, including signaling kinases and neurotransmitter receptors (1–4). In particular, STEP dephosphorylates specific residues on extracellular signal-regulated kinases 1 and 2 (ERK1/2) (2, 5), stress-activated protein kinase p38 (p38) (4, 6), and proline-rich tyrosine kinase 2 (Pyk2) (3). Moreover, STEP modulates synaptic plasticity by promoting the internalization of N-methyl-D-aspartate receptors (NMDARs) and a-amino-3-hydroxy-5-methyl-4isoxazolepropionic acid receptors (AMPARs) (1, 6), thereby weakening synaptic strength and facilitating long-term depression (LTD). In addition, STEP overexpression can directly dephosphorylate SPIN90, leading to cofilin activation, depolymerizing actin, resulting in spine collapse and consequent memory impairment (7, 8).

While most of the research conducted so far has focused on understanding the function of STEP at the postsynaptic level, STEP has also been found in presynaptic terminals of dopaminergic neurons when first identified by Lombroso and colleagues (9). Consistent with this observation, Bosco et al. later confirmed STEP expression in synaptosomal preparations from adult mouse brains (10). Furthermore, hippocampal neurons from STEP knockout (STEP KO or STEP^−/−^) animals show increased excitatory synaptic strength, associated with disrupted intracellular Ca^2+^ homeostasis and an enlarged readily releasable pool of synaptic vesicles (10). However, the precise function of STEP at the presynaptic level remains largely elusive, particularly it is currently unknown if STEP regulates presynaptic terminal differentiation during neuronal development.

Interestingly, STEP expression is dysregulated in several neurodevelopmental disorders, for which abnormal synaptic development has been reported. For example, in *Fmr1* KO mice, which present defects in presynaptic terminal formation (11–13), STEP levels are elevated (14, 15). Notably, genetic or pharmacological reduction of STEP activity rescues behavioral, electrophysiological, and dendritic spine abnormalities in *Fmr1* KO mice, indicating that increased STEP levels contribute to some of the core features of Fragile X syndrome (FXS) (14, 15). Together, these findings strengthen the hypothesis that STEP plays a crucial function in the formation and maturation of the presynaptic terminal.

In this study, we aimed to uncover the role of STEP in presynaptic differentiation and synapse formation during neuronal development. Using genetic and pharmacological approaches, together with a reconstructed circuit-on-a-chip model, we investigated how STEP loss shapes presynaptic assembly and hippocampal synapse density. Mechanistically, we show that developmental inactivation or deletion of STEP promotes presynaptic bouton maturation by relieving a phosphatase brake on synaptic vesicle clustering and release. Through functional imaging and multielectrode array recordings, we examined how STEP deletion enhances vesicle release efficacy and elevated network activity. We further explored whether selective presynaptic inhibition of STEP activity is sufficient to drive synaptogenesis, and if it rescues presynaptic abnormalities in *Fmr1* KO neurons. Together, these findings identify STEP as a developmental brake on presynaptic differentiation and suggest that targeting STEP may represent a promising strategy to ameliorate synaptic dysfunction in Fragile X Syndrome.

## Results

### STEP Is Present in Axonal/Presynaptic Compartments During Early Neuronal Development.

**Fig. 1.**
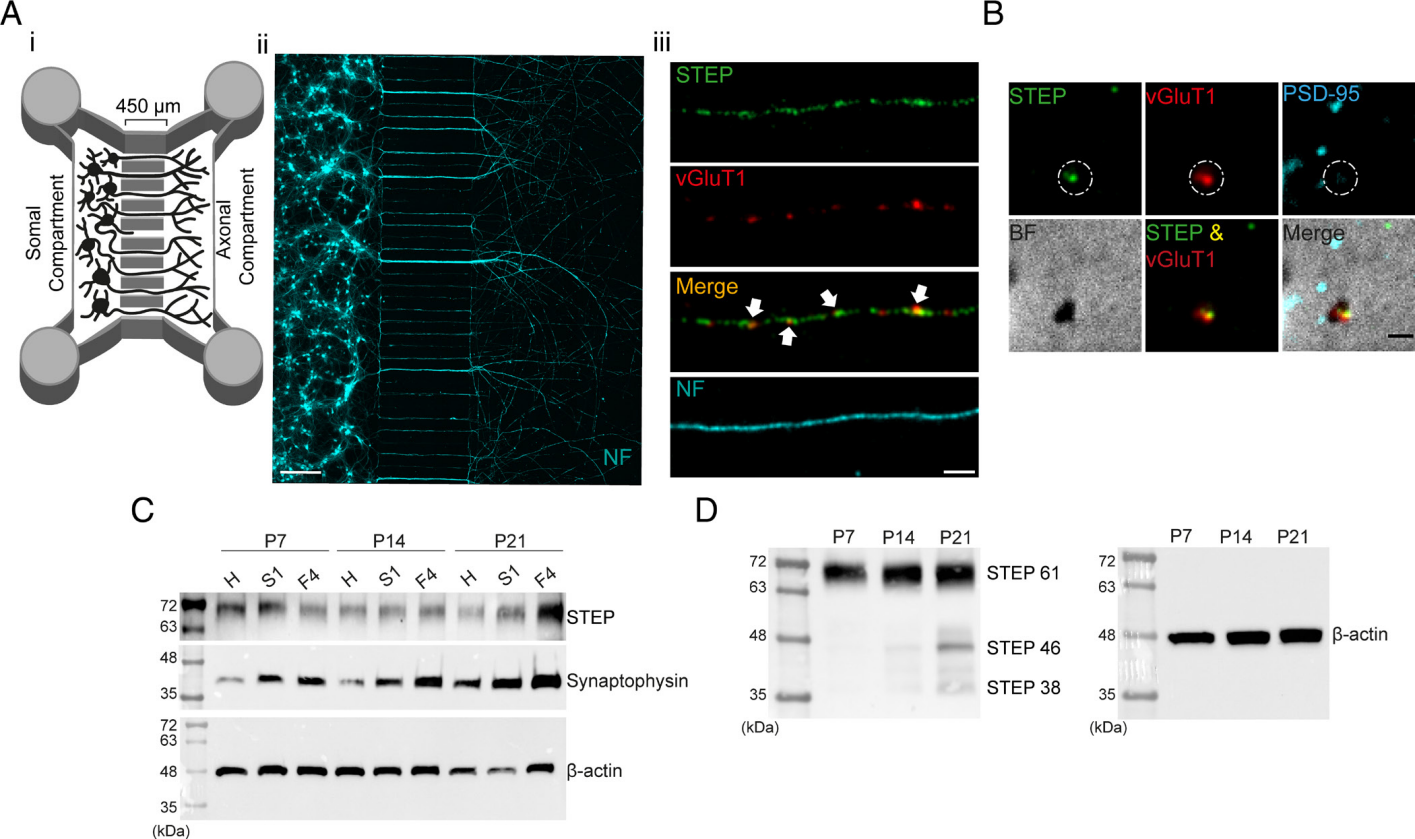
STEP is present in axonal compartments and associated with presynaptic terminals during early neuronal development. (*A*) STEP colocalizes with the presynaptic marker VGluT1 in glutamatergic axons. Rat embryonic hippocampal neurons were cultured in microfluidic chambers for axonal isolation and analyzed at DIV8. (*i*) Schematic representation of the microfluidic chamber. The device consists of a somal compartment and an axonal compartment (each 1.5 mm wide and 7 mm long) separated by microgrooves (450 μm long, 10 μm wide). (*ii*) Representative image of hippocampal neurons cultured in microfluidic chambers, showing that cell bodies are restricted to the somal compartment, while only axons extend into the opposite compartment [cyan, neurofilament (NF) labeling]. (Scale bar, 200 μm.) (*iii*) Axons immunostained against STEP (green), the presynaptic protein VGluT1 (red), and the axonal marker neurofilament (NF, cyan). STEP is present in distal axons of hippocampal neurons and colocalizes with VGluT1, as indicated by white arrowheads. (Scale bar, 2.5 μm.) (*B*) STEP immunoreactivity in purified synaptosomes. Synaptosomes were prepared from the hippocampus of postnatal mouse pups at day 7. STEP (green) is found to colocalize with the presynaptic maker VGluT1 (red) in the isolated synaptosomal fraction (Fraction 4). BF-bright field. (Scale bar, 0.5 μm.) (*C*) Validation of synaptosome preparation using a Percoll gradient. Representative immunoblots showing STEP expression and enrichment of synaptophysin in homogenate (H), supernatant (S1), and purified synaptosomes (Fraction 4, F4) isolated from the mouse hippocampus at P7, P14, and P21. (*D*) Representative immunoblots of STEP expression in synaptosomes (Fraction 4) isolated from the rat hippocampus at different stages of neuronal development (P7, P14, and P21).

Although most of the studies focus on the postsynaptic role of STEP, this protein has also been found in presynaptic terminals of dopaminergic neurons (9), and more recently in synaptosomes isolated from the brains of adult mice (10). To directly test whether STEP is present in developing hippocampal axons, we analyzed its distribution in neurons cultured in microfluidic chambers, which physically separate axons from cell bodies and dendrites and thereby enable selective analysis of isolated axons (Fig. 1*A*). Neurons were immunostained against neurofilament (axonal marker), Vesicular glutamate transporter 1 (VGluT1, a presynaptic marker of glutamatergic synaptic vesicles) and STEP, using an anti-STEP-specific antibody (*SI Appendix*, Fig. S1*A*). STEP immunoreactivity was observed along isolated axons, with a subset of STEP puncta colocalizing with VGluT1 puncta, indicating its presence in axonal/excitatory presynaptic terminals during early stages of neuronal development (Fig. 1*A*).

To further confirm STEP presence at the presynaptic level, we performed immunocytochemistry on purified hippocampal synaptosomes from postnatal day 7 (P7) rats. STEP colocalizes with the presynaptic marker VGluT1, supporting its localization in the presynaptic region (Fig. 1*B*). Western blot analysis of hippocampal homogenates and synaptosomal fractions collected at P7, P14, and P21 revealed STEP immunoreactivity in both total homogenates and isolated synaptosomes (Fig. 1*C*). STEP levels were particularly enriched in Fraction 4, the purified synaptosomal fraction, at P14 and P21 (Fig. 1*C*). Interestingly, additional STEP isoforms of approximately 38 and 46 kDa are apparent in the purified synaptosomal fraction (Fig. 1*D*). Their specific roles in synaptic maturation and neuronal circuit formation during early neurodevelopment are unexplored and should be the focus of future studies. Together, these data demonstrate that STEP is expressed at presynaptic sites at the early stages of neuronal development.

### Presynaptic Differentiation Is Regulated by STEP in Developing Axons of Hippocampal Neurons.

**Fig. 2.**
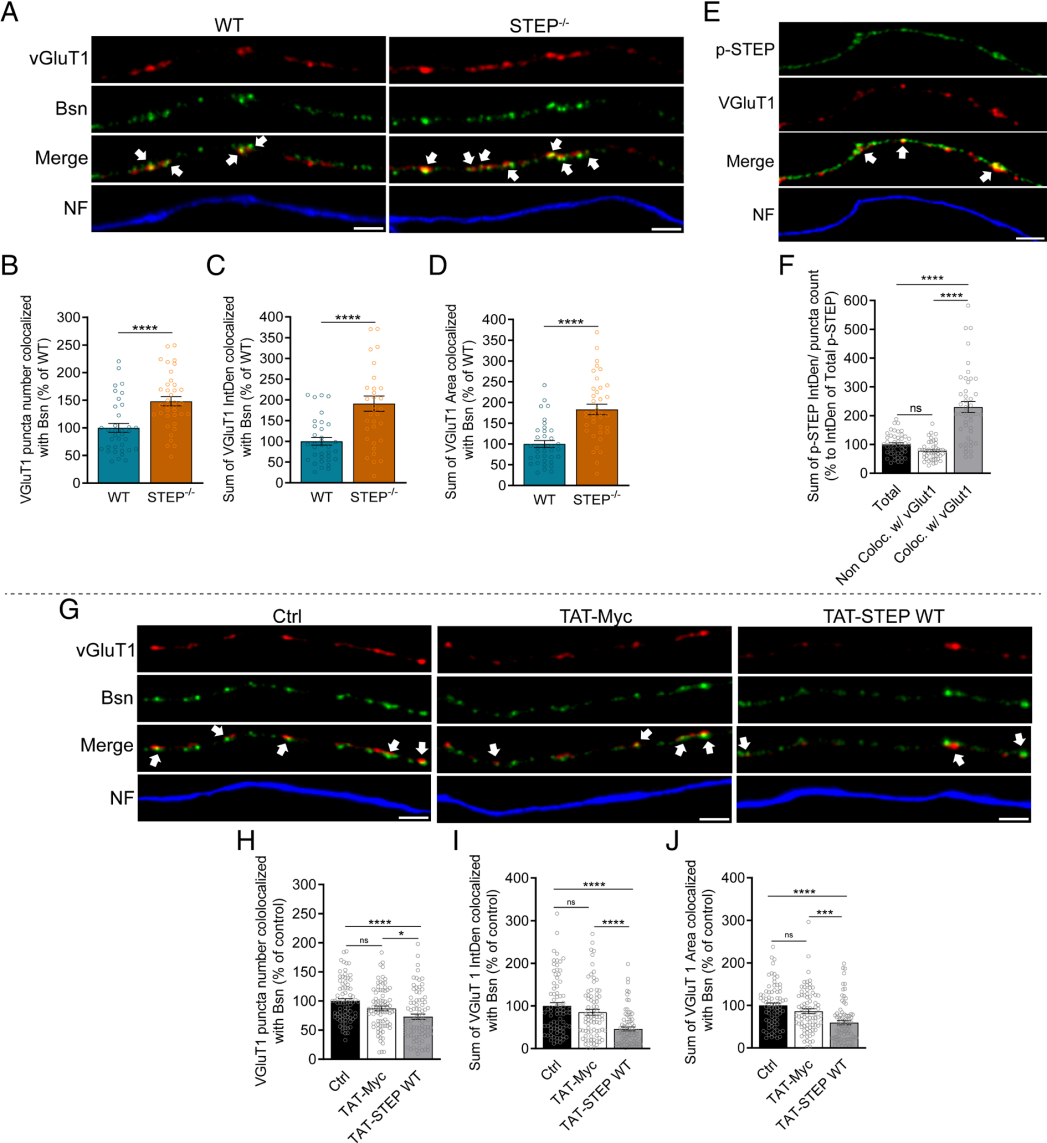
Presynaptic differentiation is regulated by STEP in developing axons of hippocampal neurons. (*A*) Genetic deletion of STEP increases presynaptic differentiation in developing axons. Hippocampal neurons from WT and STEP^−/−^ mice grew as pseudoexplants until DIV9, and the formation of presynaptic clusters was assessed by immunocytochemistry using antibodies against the synaptic vesicle marker VGluT1 (red) and the presynaptic active zone marker Bassoon (green). Neurofilament (blue) was used as an axonal marker. STEP deletion induced an increase in presynaptic differentiation of hippocampal neurons. White arrowheads show presynaptic clusters along the axon. (Scale bar, 2.5 μm.) (*B*–*D*) Quantitative data of number of presynaptic clusters per axonal length, hereby considered as clusters in which VGluT1 punctum (SV marker) and Bassoon punctum larger than 0.05 µm^2^ (active zone marker) colocalize. Results are expressed as % of the values obtained in WT neurons. Bars represent the mean ± SEM of 35 (WT) and 37 (STEP^−/−^) fields of view from 3 independent experiments. (*E* and *F*) Increased STEP inactivation at presynaptic terminals in developing axons of hippocampal neurons. Hippocampal neurons were cultured as pseudoexplants until DIV9 and then immunostained against the phosphorylated form of STEP (p-STEP; green), the vesicular glutamate transporter (VGluT1; red), and the axonal marker neurofilament (NF; blue). White arrowheads indicate that p-STEP, the inactive form of STEP, is localized at presynaptic sites. (Scale bar, 2.5 μm) (*E*). Quantification of the integrated density of p-STEP puncta colocalized or not with VGluT1 per total puncta count, shows that inactive STEP is enriched at glutamatergic presynaptic sites. Data are expressed as % to the integrated density of total p-STEP integrated density. Bars represent the mean ± SEM of 46 fields of view from 3 independent experiments (*F*). (*G*) STEP overexpression decreases presynaptic differentiation in developing axons of hippocampal neurons. Pseudoexplants of hippocampal neurons were incubated at DIV9 with 2 µM TAT-based membrane-permeable peptides: a constitutively active form of STEP (TAT-STEP WT) and the control TAT-Myc. After 1 h, clustering of presynaptic material was assessed by immunostaining against the synaptic vesicle marker VGluT1 (red) and the presynaptic active zone marker Bassoon (green). Neurofilament (blue) was used as an axonal marker. TAT-STEP WT decreases the clustering of presynaptic material when compared to nontreated control neurons (Ctrl), indicating that increased levels of STEP lead to a reduction in presynaptic differentiation. White arrowheads show presynaptic clusters along the axon. (Scale bar, 2.5 μm.) (*H–J*) Number of presynaptic clusters per axonal length, hereby considered as clusters in which a VGluT1 punctum (SV marker) and a Bassoon punctum larger than 0.05 µm^2^ (active zone marker) colocalize. Results are expressed as % of control neurons. Bars represent the mean ± SEM of 39 (Ctrl), 44 (TAT-Myc), and 47 (TAT-STEP WT) fields of view from 3 independent experiments. (*B*–*D, F* and *H*–*J*) Statistical significance was assessed by the nonparametric Mann–Whitney test with ****P < 0.0001 (*B*–*D*) and by the Kruskal–Wallis test followed by Dunn’s multiple comparison test with ****P < 0.0001; ***p < 0.001; *P < 0.05; ns = not significant, *P* > 0.05 (*F* and *H*–*J*).

To determine whether genetic deletion of STEP modulates presynaptic differentiation, we cultured mouse embryonic hippocampal neurons from WT and STEP^−/−^ mice as pseudoexplants (*SI Appendix*, Fig. S2). It was previously reported that STEP^−/−^ mice exhibit increased phosphorylation of STEP substrates such as ERK1/2, as well as increased surface expression of the GluN1/GluN2B receptor complex, without major disruptions to the cytoarchitecture of brain regions that typically express STEP (2, 16, 17). Here, the effect of STEP knockout on presynaptic cluster formation was assessed by analyzing the number of VGluT1 puncta colocalized with the presynaptic active zone protein Bassoon (VGluT1/Bassoon clusters), as well as the integrated density and area of VGluT1 colocalized with Bassoon per unit of axonal length (Fig. 2*A*). We found that the genetic deletion of STEP increases VGluT1/Bassoon presynaptic clustering in developing axons of hippocampal neurons (Fig. 2*B*). Robust increases were also observed for the puncta integrated density (Fig. 2*C*) and area (Fig. 2*D*), indicating that STEP regulates presynaptic differentiation. To examine whether phosphorylated STEP (p-STEP), the inactive form of STEP, is localized at presynaptic sites, hippocampal pseudoexplants were immunostained for phospho-STEP, VGluT1, and the axonal marker neurofilament (Fig. 2*E*). p-STEP immunoreactivity was observed along axons and frequently overlapped with VGluT1 puncta, indicating its presence at excitatory presynaptic terminals. Quantitative analyses revealed that the integrated density of p-STEP signal colocalized with VGluT1 was significantly higher than the noncolocalized fraction (Fig. 2*F*). These results show that inactive STEP is enriched at glutamatergic presynaptic sites.

We next examined whether STEP overexpression has the opposite effect on presynaptic differentiation. To test this, we enhanced STEP activity by administering a constitutively active form of STEP (TAT-STEP WT) to rat embryonic hippocampal pseudoexplants. The transactivator of transcription (TAT) sequence (18) was fused to STEP to generate the cell-permeable TAT-STEP WT protein, which was additionally tagged with a Myc epitope. An inactive TAT-Myc peptide was used as a control (19). Immunocytochemical detection of the Myc epitope confirmed comparable neuronal uptake and intracellular accumulation of both constructs (*SI Appendix*, Fig. S3 *A* and *B*). As expected, TAT-Myc showed a predominantly diffuse intracellular distribution, whereas TAT-STEP WT exhibited a more punctate pattern in the soma and neurites (*SI Appendix*, Fig. S3*A*). TAT-STEP WT but not the TAT-Myc peptide, reduced the number of VGluT1/Bassoon presynaptic clusters (Fig. 2 *G* and *H*). Moreover, we also found a pronounced reduction in the integrated density and area of VGluT1 puncta that colocalized with Bassoon (Fig. 2 *I* and *J*). Overall, these results suggest that STEP acts as a repressor of presynaptic bouton formation during early neuronal development, as its genetic deletion leads to an increase in presynaptic differentiation, whereas STEP overexpression has the opposite effect.

### Genetic Deletion of STEP Enhances Synapse Formation at Early Stages of Neuronal Development.

**Fig. 3.**
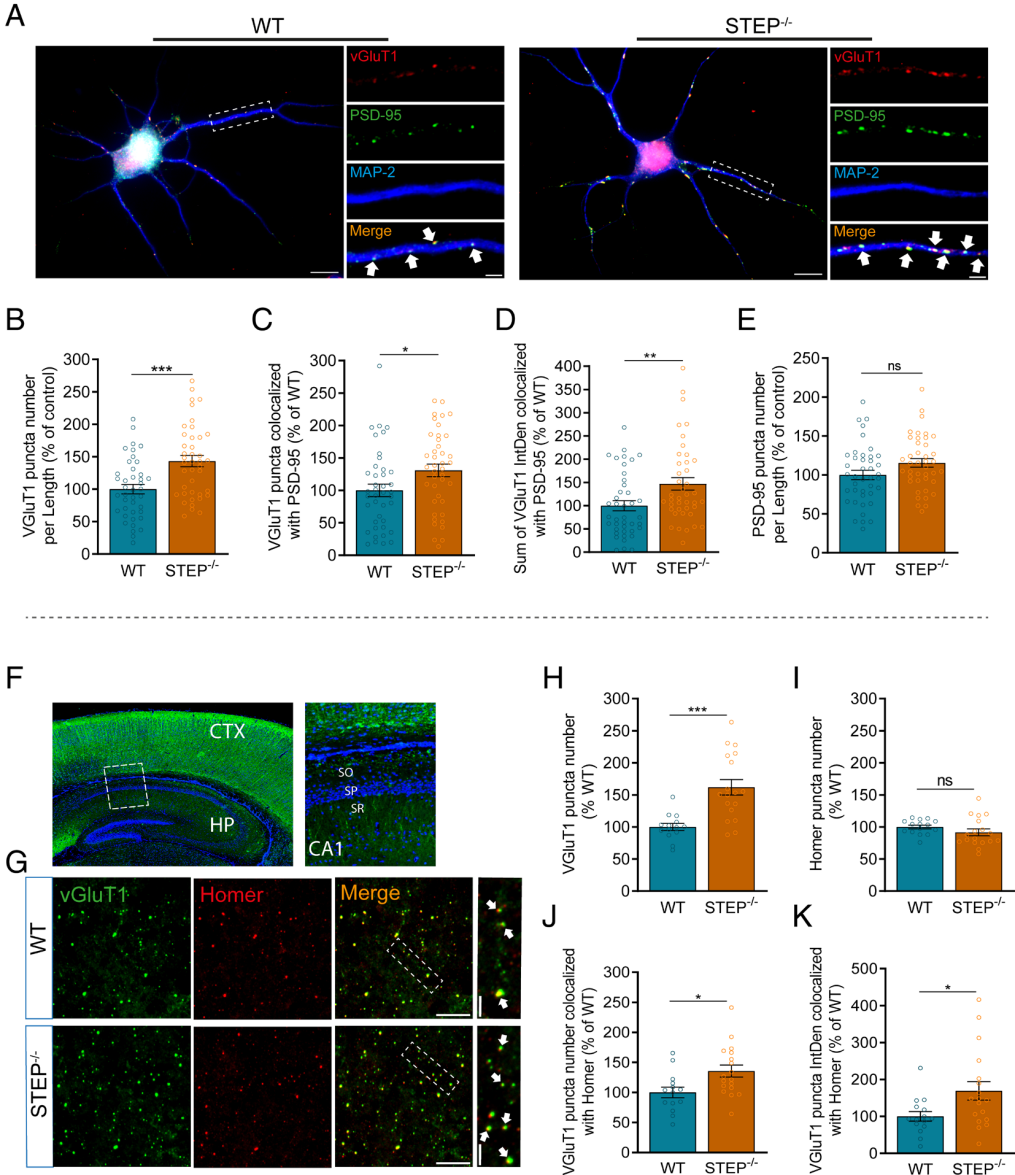
Hippocampal axodendritic synapse formation is enhanced in STEP^−/−^ mice in vitro and in vivo. (*A*) STEP^−/−^—derived cultures present increased axodendritic synapse density. Low-density hippocampal neurons were immunostained at DIV9 against the presynaptic marker VGluT1 (red), the postsynaptic marker PSD-95 (green) and the somatodendritic marker MAP-2 (blue). Genetic deletion of STEP increases the number of VGluT1 puncta, and increases the number of axodendritic synapses as observed by an increase in the number of VGluT1/PSD-95 clusters. Notably, VGluT1 puncta that colocalize with PSD-95 have increased integrated density. No differences were seen for PSD-95 puncta number. White arrowheads show VGluT1/PSD-95 puncta colocalization along the dendrite. [Scale bars, 10 μm (*Insets*, 2.5 μm).] (*B*–*E*) Number of VGluT1 puncta per dendritic length expressed as % of WT neurons (*B*). VGluT1 puncta number (*C*) and integrated density (*D*) that colocalize with PSD-95 puncta per dendritic length expressed as % of values presented in WT neurons. Quantitative data of PSD-95 puncta number (*E*) per dendritic length expressed as % of WT neurons. Bars represent the mean ± SEM of 39-41 fields of view from 3 independent experiments. (*F*) Photomicrograph of a coronal brain section of postnatal day 9 (PN 9) mice showing the cortex (CTX) and hippocampus (HP) regions. The slice was immunolabeled with the dendritic marker MAP-2 (green), and nuclei were stained with Hoechst 33342 (blue). The insets of dash box in the left image depict the hippocampal stratum oriens (SO), stratum pyramidalis (SP), and stratum radiatum (SR) layers of CA1 hippocampal region. (*G*) STEP^−/−^ mice have increased synapse density in CA1 hippocampal region. Representative images of stratum oriens from PN9 WT and STEP^−/−^ mice stained for the presynaptic marker VGluT1 (green) and the postsynaptic marker Homer (red). Genetic deletion of STEP increases the number of presynaptic puncta and, importantly, increases the number of axodendritic synapses as observed by an increase in the number of VGluT1/Homer clusters. This indicates that STEP deletion increases the number of synapses in vivo. The arrows in the merged channel of insets indicate colocalized puncta. [Scale bar, 10 μm (*Insets*, 2.5 μm).] (*H*–*K*) Quantitative data of VGluT1 (*H*) and Homer (*I*) individual puncta number, VGluT1/Homer cluster number (*J*) and integrated density (*K*). Bars represent the mean ± SEM of 3 slices per animal (5 WT and 6 STEP^−/−^ mice). (*B*–*E* and *H*–*K*) Statistical significance was assessed by two-tailed, unpaired Student’s *t* test or nonparametric Mann–Whitney test with ***P < 0.001; **P < 0.01; *P < 0.05; ns = not significant, *P* > 0.05.

We next sought to investigate whether genetic deletion of STEP regulates axodendritic synapse formation. To this end, we used low-density hippocampal cultures from embryonic WT and STEP^−/−^ mice and immunostained against the presynaptic marker VGluT1, the postsynaptic marker PSD-95 and the somatodendritic marker MAP2 (Fig. 3*A*). STEP^−/−^ cultures showed an increased number of VGluT1 puncta (Fig. 3*B*). Importantly, genetic deletion of STEP increased the number of VGluT1 puncta colocalized with PSD-95 (Fig. 3*C*). Likewise, the integrated density of VGluT1 puncta that colocalized with PSD-95 was also increased (Fig. 3*D*). Interestingly, no significant changes were found in the number of PSD-95 puncta (Fig. 3*E*), suggesting that presynaptic STEP modulates axodendritic synapse formation.

To determine whether STEP regulates synapse formation in vivo, we investigated if STEP^−/−^ mice display altered synapse density in the CA1 hippocampal region. Coronal sections of the hippocampus from postnatal day 9 (P9) mice were immunostained for the presynaptic marker VGluT1 and the postsynaptic marker Homer. Since most STEP immunoreactivity in the hippocampus was found in CA2 region (9, 20) which projects extensive axonal arbors to the stratum oriens layer of the CA1 region (21, 22), we quantified synapse density in the stratum oriens layer (Fig. 3 *F* and *G*). We found that genetic deletion of STEP increases the number of VGluT1 puncta (Fig. 3*H*) and, importantly, in STEP^−/−^ mice, we observed an increase in the number of puncta showing VGluT1/Homer colocalization (Fig. 3*J*), as well as an increase in the intensity of these puncta (Fig. 3*K*). On the other hand, no statistically significant changes were found in Homer puncta number (Fig. 3*I*). Collectively, these data show that STEP regulates synapse formation both in vitro and in vivo.

### A Circuit-on-a-Chip Reveals the Contribution of Presynaptic STEP to Synapse Formation.

**Fig. 4.**
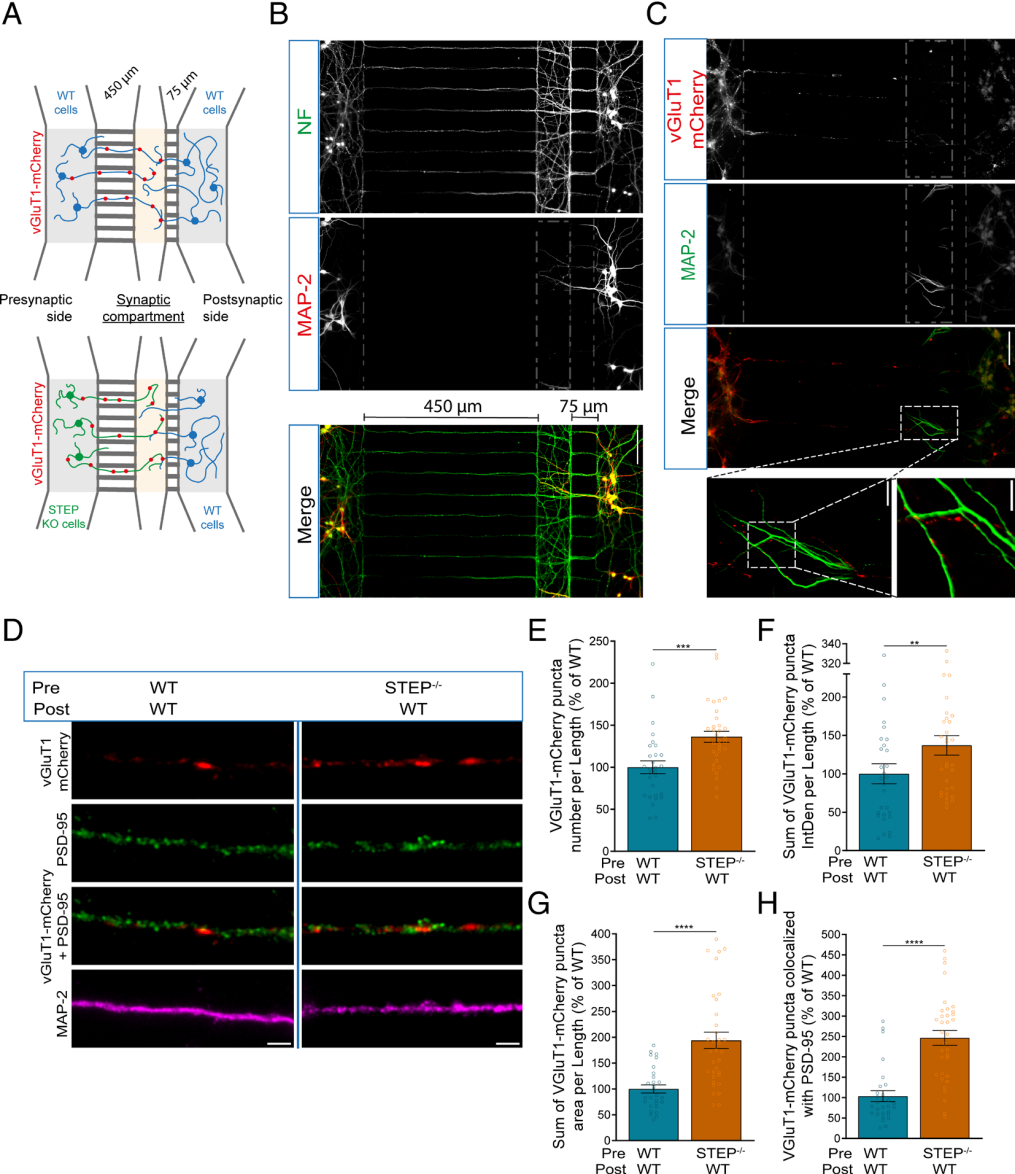
Circuit-on-a-chip data reveals the essential contribution of presynaptic STEP to synapse formation. (*A*) Schematic tripartite microfluidic device designed to compartmentalize axodendritic synapses. In the synapse formation chamber, three compartments are delineated by two sets of microchannels with differing lengths. The presynaptic and postsynaptic compartments are linked to the central synaptic compartment by microchannels measuring 450 µm and 75 µm, respectively. Owing to the shorter length of the second set of microchannels, dendrites from the postsynaptic compartment extend into the synaptic compartment, where they contact axons from the presynaptic compartment. Cylinders represent accessible wells for seeding neurons and/or perfusing liquids (culture medium and viral particles). (*B* and *C*) Photomicrographs of the circuit-on-a-chip. (*B*) Axons (green), but not dendrites (red), from the presynaptic compartment cross the 450 µm microchannels to reach the central synaptic compartment. Conversely, dendrites from the postsynaptic compartment reach the central synaptic compartment by crossing the 75 µm microchannels. (Scale bar, 100 μm.) (*C*) Within the central synaptic compartment, axons originating from the presynaptic compartment were distinguished from those originating from the postsynaptic compartment by transduction with VGluT1-mCherry viral particles (red). (Scale bar, 100 μm.) Insets show axons that contact with dendrites (green). (Scale bar 10 μm and 5 μm.) (*D*) Synapses number increases between STEP^−/−^ axons and WT dendrites. At DIV6, neurons in the presynaptic compartment were transduced with VGluT1-mCherry viral particles. After 72 h neurons were immunostained for mCherry (red), the postsynaptic marker PSD-95 (green) and the somatodendritic marker MAP-2 (magenta). Genetic deletion of STEP increases the number, integrated density, and area of VGluT1-mCherry puncta, and notably increases the colocalization of VGluT1mCherry puncta with PSD-95 puncta. Results demonstrate an increase in the number of axodendritic synapses when STEP^−/−^ axons contact with STEP^+/+^ dendrites, when compared with WT–WT synapses. (Scale bar, 2.5 μm.) (*E*–*H*) Quantification of VGluT1mCherry puncta number (*E*), integrated density (*F*), area (*G*), and VGluT1-mCherry/PSD-95 puncta colocalization (*H*) per dendritic length. Bars represent the mean ± SEM of 28-34 transduced axons overlapping dendritic segments from 3 independent experiments. Data are expressed as % of the values obtained with the WT–WT circuit configuration. Statistical significance was assessed by the nonparametric Mann–Whitney test with ****P < 0.0001, ***P < 0.001 and **P < 0.01.

To further dissect the contribution of presynaptic STEP to axodendritic synapse formation, we reconstructed an in vitro hippocampal network system using microfluidic devices (Fig. 4*A*). These devices allow independent access and manipulation of synapses and their pre- and postsynaptic compartments (23, 24). In this system, axons but not dendrites, from the presynaptic compartment cross the long microgrooves (450 µm) into the middle compartment, while neurons from the postsynaptic compartment project both axons and dendrites into the middle compartment (Fig. 4*B*). Axons and dendrites form synapses in the middle compartment, which can be analyzed using fluorescence microscopy. Two distinct circuits-on-a-chip were established to specifically determine the contribution of presynaptic STEP to synapse formation. We established one circuit between WT presynaptic and WT postsynaptic neurons (WT–WT) and another circuit between STEP^−/−^ presynaptic neurons and WT postsynaptic neurons (STEP^−/−^–WT) (Fig. 4*A*). Additionally, STEP^−/−^ and WT neurons in the presynaptic compartment of microfluidic devices were transduced with VGluT1–mCherry lentiviral particles to distinguish the axons originating from the presynaptic compartment from those originating from the postsynaptic compartment (Fig. 4*C*). Neurons were immunostained against mCherry (indicating the presynaptic marker VGluT1) and the postsynaptic marker PSD-95 (Fig. 4*D*). We found that axons from STEP^−/−^ neurons displayed an increased number of VGluT1–mCherry puncta (Fig. 4*E*), as well as higher integrated density (Fig. 4*F*), and larger area (Fig. 4*G*). Importantly, the genetic deletion of presynaptic STEP greatly increased the number of VGluT1/PSD-95 clusters between STEP^−/−^ axons and WT dendrites, compared with the WT–WT circuit (Fig. 4*H*). Taken together, these results show that STEP functions as a negative regulator of presynaptic differentiation during neuronal development.

### STEP Deletion Enhances Presynaptic Vesicle Release and Promotes Coordinated Neuronal Network Activity.

**Fig. 5.**
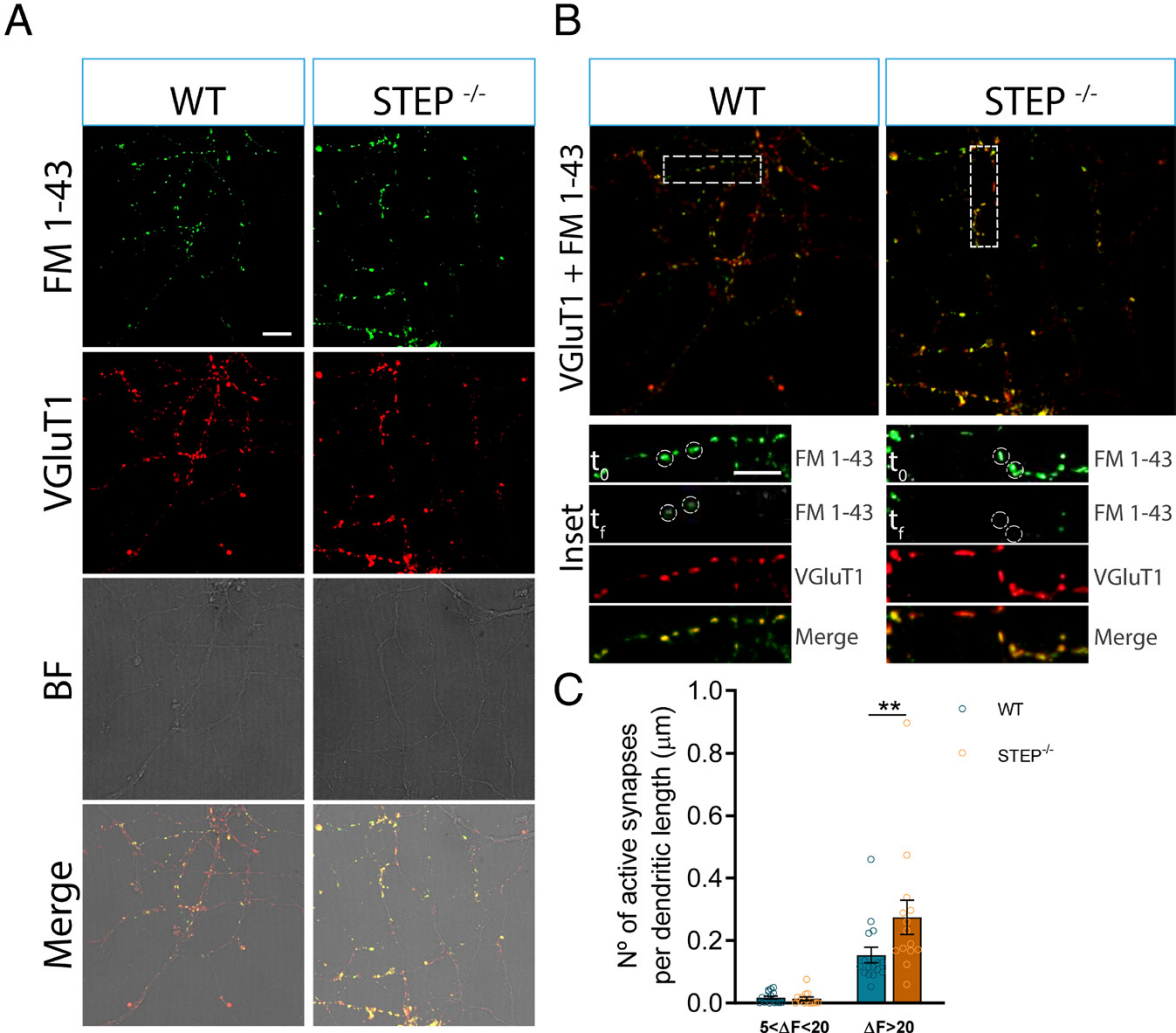
Genetic deletion of STEP increases the number of active synapses. (*A*) Representative images of WT and STEP^−/−^ hippocampal neuronal cultures stained with FM1-43 (green) and VGluT1 (red), showing synaptic vesicle release and glutamatergic synapse localization, respectively. Merged images demonstrate colocalization of FM1-43 and VGluT1 puncta, indicative of active glutamatergic synapses. (Scale bar, 20 μm.) (*B*) Insets show time-lapse FM1-43 imaging of individual synapses before (t^0^) and after (t_f_) depolarization with 50 mM KCl. Circles indicate synapses that show FM1-43 fluorescence loss, identifying them as active. (*Insets* Scale bar, 10 μm.) (*C*) Quantification of the number of active synapses, measured 60 s poststimulation with 50 mM KCl, per unit dendritic length (μm) in WT (blue) and STEP^−/−^ (orange) neurons, categorized by FM1-43 fluorescence loss: moderately functional synapses (5 < ΔF < 20) and highly active synapses (ΔF > 20). STEP^−/−^ neurons show a significantly higher number of highly active synapses compared to WT. Bars represent the mean ± SEM of 14 to 16 fields of view from 4 independent. Statistical significance was assessed by the two-way ANOVA followed by Bonferroni post hoc with **P < 0.01 analysis.

To determine whether the increased number of synapses observed in STEP^−/−^ mice are functionally active rather than immature or nonfunctional (“silent”) ones, we assessed synaptic vesicle release using FM1-43 dye-based assay, routinely used for the quantification of synaptic vesicle recycling (24). To specifically identify excitatory presynaptic boutons, cultures were transduced with VGluT1–mCherry lentiviral particles, which allowed us to correlate structural puncta with FM1-43-based measures of vesicle recycling. Importantly, in this analysis, active synapses were defined as VGluT1-positive puncta that exhibit FM1-43 destaining upon depolarization, thus integrating both structural and functional criteria. Functional synapses were then categorized into two groups based on the magnitude of fluorescence loss (ΔF) following 60 s of KCl-induced depolarization: moderately active synapses (5% < ΔF < 20%) and highly active synapses (ΔF > 20%). Although the thresholds used here to distinguish moderately and highly active synapses are arbitrary, a similar approach was used by Henkel et al., who categorized synapses based on ΔF/F_max_ following FM dye destaining (25). Using this classification, we found that while the proportion of moderately active synapses was comparable between genotypes, DIV9 hippocampal STEP^−/−^ cultures exhibited a significant increase in the number of highly active synapses (Fig. 5 *A*–*C*). This indicates that STEP deletion not only leads to the formation of more synapses, but that these synapses exhibit enhanced presynaptic performance.

**Fig. 6.**
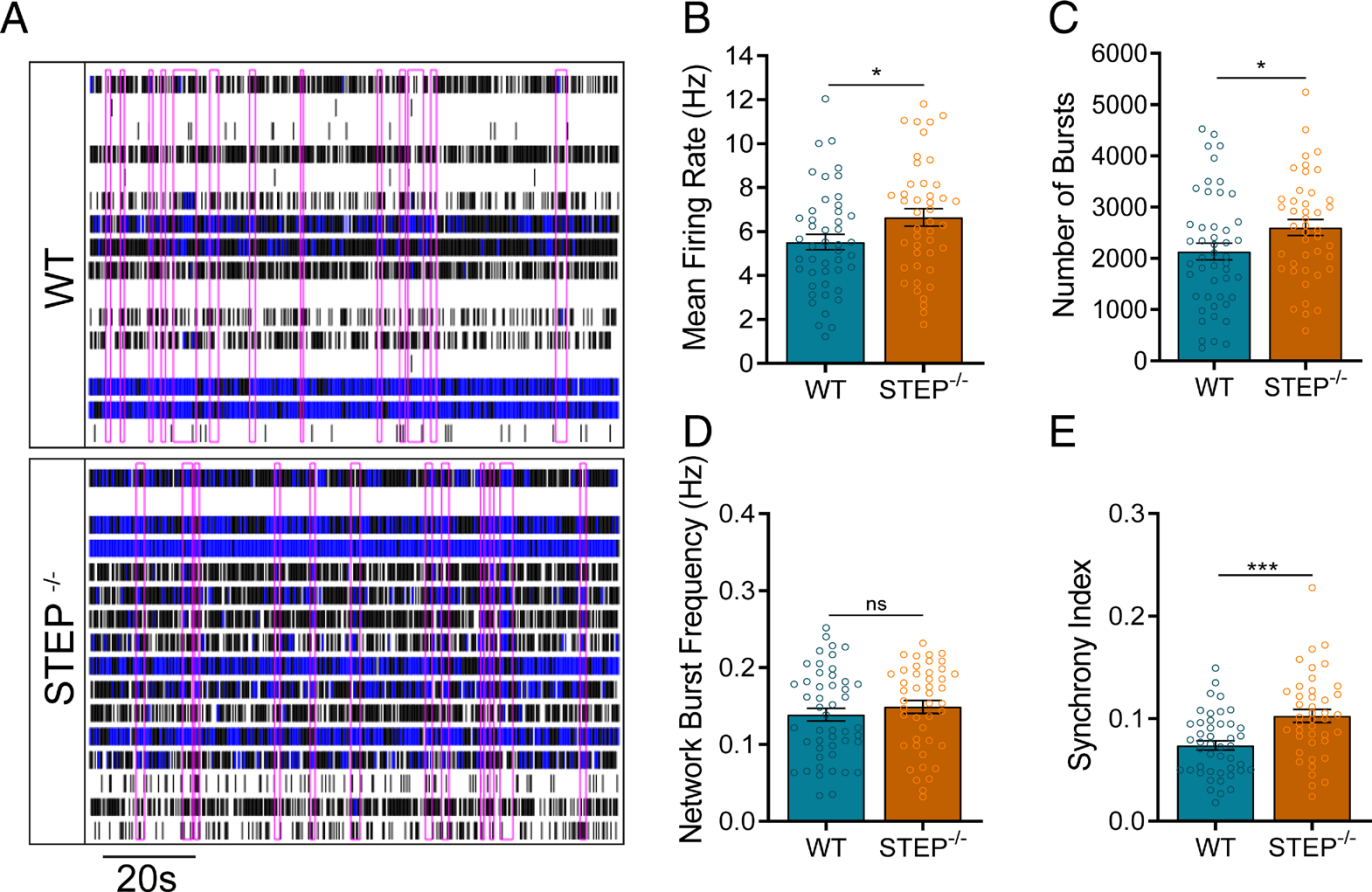
Genetic deletion of STEP increases hippocampal neuronal excitability. (*A*) Representative raster plots of multielectrode array analysis displaying 120 s of spontaneous activity from WT and STEP^−/−^ mice embryonic hippocampal neurons at DIV14. Spikes are represented in black, spike bursts in blue and network bursts are highlighted by magenta rectangles. Every line on each raster plot corresponds to single-electrode recordings (16 electrodes per well). (*B*–*E*) Quantification of extracellular recordings in WT and STEP^−/−^ cultures. Results show increased Mean Firing Rate (*B*), Number of bursts (*C*), and Synchrony Index (*E*), without significant changes in Network Burst Frequency (*D*) in STEP^−/−^ cultures compared to WT. These findings indicate that STEP inactivation promotes functional presynaptic maturation. Bars represent the mean ± SEM of 34 (WT) and 32 (STEP^−/−^) wells from 3 independent experiments. Statistical significance was assessed by the nonparametric Mann–Whitney test with ***P < 0.001; *P < 0.05; ns = not significant, *P* > 0.05.

To determine whether the enhanced synapses observed have a functional impact on neuronal network activity, we used multielectrode arrays. This technology allows noninvasive and long-term recording of neuronal culture activity at the network level (26, 27). We performed extracellular recordings from hippocampal WT and STEP^−/−^ cultures at DIV9. Raster plots (Fig. 6*A*) showed that STEP^−/−^ cultures had increased Mean Firing Rate (Fig. 6*B*), number of bursts (Fig. 6*C*), and higher synchrony index (Fig. 6*E*) compared to WT cultures, indicating greater overall excitability and improved temporal coordination within the network. Notably, network burst frequency remained unchanged (Fig. 6*D*), suggesting that while the global rate of large-scale network events did not differ, the ongoing activity was more synchronous and efficiently propagated in STEP^−/−^ networks. Together, these findings show that deletion of STEP during early development promotes the formation of functional synapses, which in turn enhances neuronal network excitability and synchrony.

### STEP Inactivation Reverses Presynaptic Abnormalities in *Fmr1* KO Hippocampal Neurons.

**Fig. 7.**
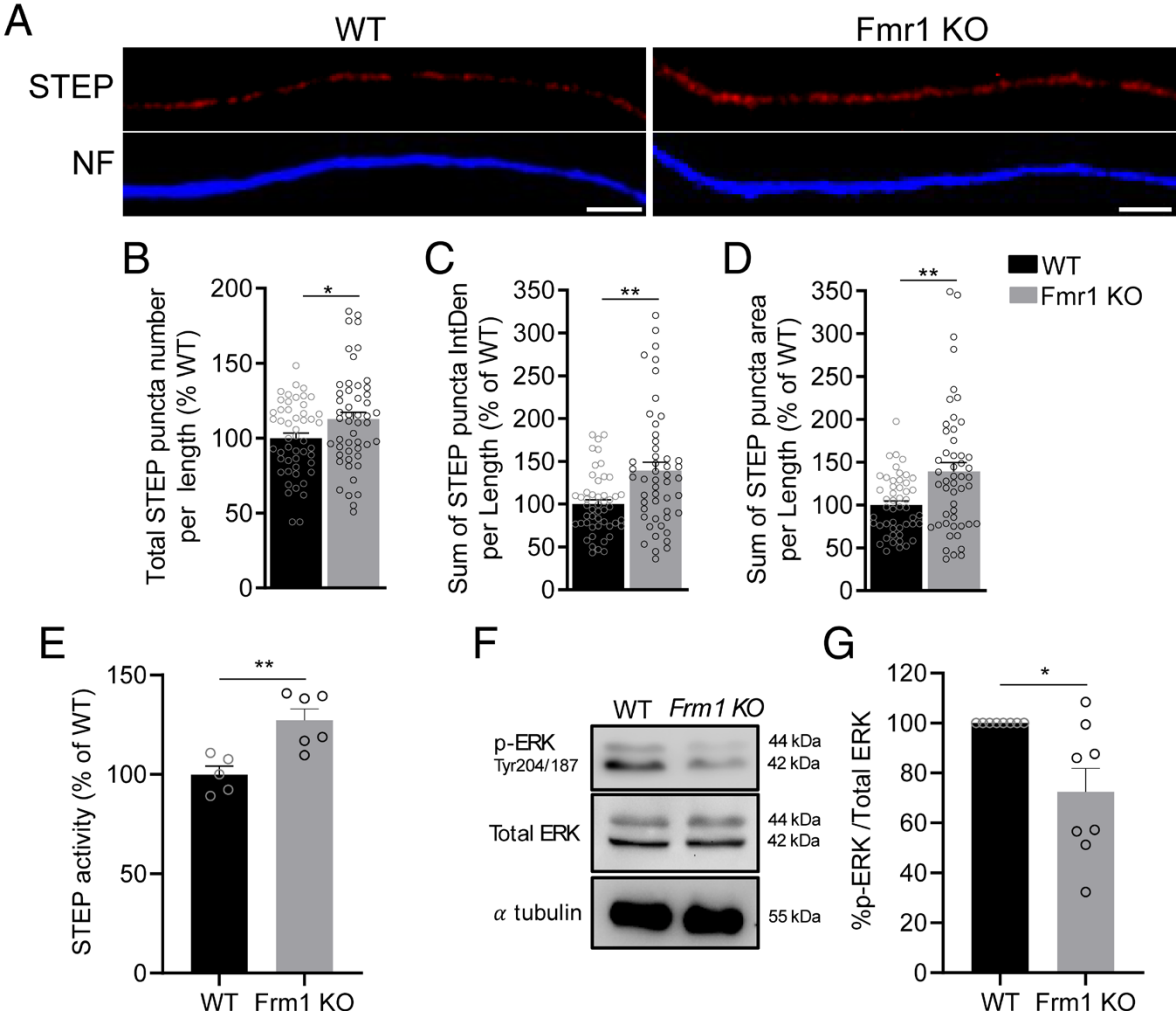
Presynaptic STEP is upregulated and functionally active in *Fmr1* KO hippocampal neurons. (*A*) Example of hippocampal axons from WT and *Fmr1* KO mice, note increased STEP levels in *Fmr1* KO axons. Hippocampal neurons from WT and *Fmr1* KO mice were grown as pseudoexplants until DIV12, and axonal STEP levels were assessed by immunostaining against STEP (red). Neurofilament (blue) was used as an axonal marker. Genetic deletion of *Fmr1* increases STEP puncta number, integrated density, and area along axons compared to WT axons. (Scale bar, 2.5 μm.) (*B*–*D*) *Fmr1* KO neurons display increased STEP puncta number (*B*), integrated density (*C*), and area (*D*) per axonal length, expressed as % of the values obtained in WT axons. Bars represent the mean ± SEM of 49-50 fields of view from 4 independent experiments. (*E*) STEP phosphatase activity is increased in presynaptic terminals of *Fmr1* KO mice. STEP was immunoprecipitated by a specific polyclonal antibody from solubilized synaptosomes prepared from the hippocampus of postnatal day 9 (P9) WT and *Fmr1* KO mice. Colorimetric quantification of STEP activity was performed using p-NPP as substrate. Bars represent the mean absorbance at 405 nm (± SEM), normalized to WT, from 6 biological replicates. (*F* and *G*) Phospho-ERK1/2 levels are decreased in *Frm1* KO samples. Representative immunoblots showing reduced phosphorylation of ERK1/2 (pERK1/2; Thr202/Tyr204) in synaptosomal fractions from *Frm1* KO compared to WT controls (*F*). Total ERK1/2 and α-tubulin were used as loading controls (*G*). Quantification of pERK1/2 normalized to total ERK1/2 (pERK/ERK ratio). Bars represent mean ± SEM from n = 8 biological replicates. (*B*–*D*; *E* and *G*) Statistical significance was assessed by two-tailed, unpaired Student’s *t* test or nonparametric Mann–Whitney test with **P < 0.01 and *P < 0.05 (*B*–*D*) or by two-tailed, unpaired Student’s *t* test with **P < 0.01 and *P < 0.05 (*E* and *G*).

It has been shown that STEP is altered in several neurological diseases, including neurodevelopmental disorder FXS (14, 28, 29). In fact, synaptosomes isolated from the hippocampi of *Fmr1* KO mice showed increased STEP levels, and this increase was not limited to the hippocampi, since STEP levels were also higher in whole brain extracts (14). To determine whether axonal STEP levels are altered in hippocampal neurons derived from *Fmr1* KO mice, we immunostained neurons against STEP and the axonal marker neurofilament. We found that axonal STEP levels are increased in hippocampal neurons derived from *Fmr1* KO mice (Fig. 7*A*), as indicated by the increase in STEP puncta number (Fig. 7*B*), integrated density (Fig. 7*C*), and area (Fig. 7*D*). Moreover, to assess whether the increased presynaptic STEP levels observed in *Fmr1* KO neurons are associated with enhanced STEP activity, we performed a colorimetric phosphatase assay using p-nitrophenyl phosphate (pNPP) as a substrate. The assay was conducted on STEP immunoprecipitated from crude hippocampal synaptosomes isolated from postnatal day 9 (P9) mice. Compared to WT, STEP enzymatic activity was increased in *Fmr1* KO synaptosomes (Fig. 7*E*), indicating a higher dephosphorylation potential under these conditions. Furthermore, to assess whether this increase in STEP activity had functional outcomes, we quantified the phosphorylation status of ERK 1/2, a canonical STEP substrate. Western blot analyses revealed a reduction in p-ERK1/2 (Tyr204/187) levels relative to total ERK in the *Fmr1* KO mouse synaptosomes (Fig. 7 *F* and *G*), consistent with increased STEP-mediated ERK dephosphorylation. These findings indicate that STEP is not only upregulated in expression and activity, but also functionally active in modulating downstream MAPK signaling at the synapse, further supporting the notion that STEP levels are regulated by FMRP.

**Fig. 8.**
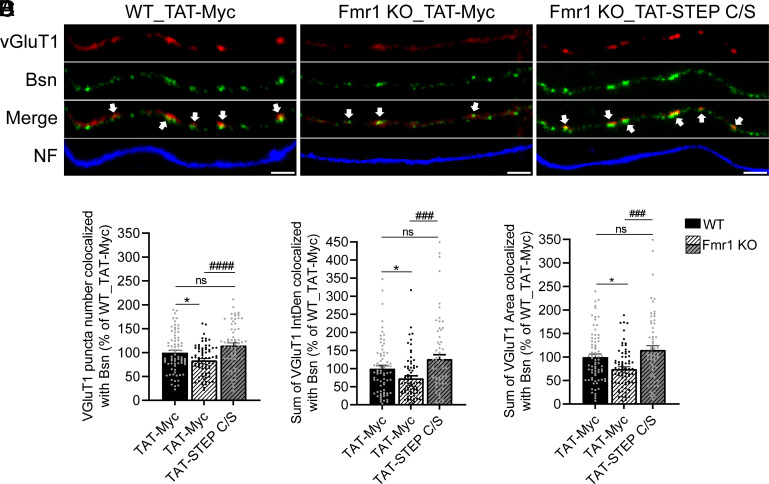
STEP inactivation reverses the presynaptic abnormalities in *Fmr1* KO hippocampal neurons. (*A*) Hippocampal neurons from WT and *Fmr1* KO mice were grown as pseudoexplants until DIV12. Cultures were incubated with 2 µM TAT-based membrane-permeable peptides: a mutated inactive form of STEP (TAT-STEP C-S) and the control TAT-Myc. After 1 h, neurons were immunostained against the synaptic vesicle marker VGluT1 (red), and the presynaptic active zone marker Bassoon (green); Neurofilament (blue) was used as an axonal marker. *Fmr1* KO axons have a reduced clustering of presynaptic material compared to WT axons, as shown by white arrowheads, indicating that genetic deletion of *Fmr1* impairs presynaptic terminals differentiation. Blocking STEP function in *Fmr1* KO neurons with the TAT-STEP C-S peptide reverses this phenotype. (Scale bar, 2.5 μm.) (*B*–*D*) Quantification of VGluT1/Bassoon puncta (*B*), integrated density (*C*), and area (*D*) of clusters per axonal length. Data are expressed as % of WT_TAT-Myc axons. Bars represent the mean ± SEM of 61-76 fields of view from 5 independent experiments. Statistical significance was assessed by the Kruskal–Wallis test followed by the Dunn’s multiple comparison test or by ANOVA followed by the Tukey’s multiple comparisons test with *P < 0.05; ^####^*P* <0.0001; ^###^*P* < 0.001; ns = not significant, *P* > 0.05.

We finally examined whether inhibiting STEP activity may rescue the synaptic abnormalities observed in FXS (11–13). To achieve this goal, hippocampal neurons derived from *Fmr1* KO mice were immunostained for VGluT1, Bassoon, and the axonal marker neurofilament (Fig. 8*A*). Results show that hippocampal cultures derived from *Fmr1* KO present a deficit in presynaptic terminal assembly, indicated by a reduction in the number of VGluT1/Bassoon clusters (Fig. 8*B*). Additionally, we observed a reduction in the integrated density (Fig. 8*C*) and area (Fig. 8*D*) of VGluT1/Bassoon presynaptic clusters. Blocking STEP activity using TAT-STEP C-S, a TAT-based membrane-permeable peptide containing a point mutation in its catalytic domain (C300S) that renders STEP enzymatically inactive (29), rescues this phenotype (Fig. 8 *A*–*D*). Collectively our results support the hypothesis that the presynaptic abnormalities observed in *Fmr1* KO neurons are improved by inhibiting STEP activity, shedding light into the molecular and cellular alterations observed in FXS.

## Discussion

In this study, we identified a role for STEP in neuronal development, specifically in regulating presynaptic differentiation. Although STEP has been predominantly characterized as a postsynaptic phosphatase, previous studies have also reported its presence in axonal compartments and nerve terminals, suggesting that its function may not be restricted to postsynaptic sites (9, 10). Consistent with this, our data demonstrate that STEP is present in axonal and presynaptic compartments during early stages of neuronal development, as supported by its localization in isolated axons and its enrichment in synaptosomal fractions. Furthermore, our findings show that suppression of STEP during early neuronal development promotes synapse formation and enhances neuronal activity, and that its dysregulation may contribute to neurodevelopmental disorders such as FXS. The importance of these findings is underscored by observations that genetic deletion of STEP enhances functional presynapse formation, whereas STEP expression leads to a reduction in the number of presynaptic clusters. Additionally, using a reconstituted circuit-on-a-chip model, we further show that selective deletion of STEP in presynaptic neurons increases the number of axodendritic synapses. Moreover, inhibition of STEP rescues presynaptic abnormalities in *Fmr1* KO neurons. Interestingly, genetic deletion of STEP increases the number of VGluT1/PSD-95 clusters in vitro and VGluT1/Homer clusters in vivo, without affecting postsynaptic marker density. This indicates that STEP acts in the axonal compartment to regulate presynaptic differentiation and, ultimately, synapse formation. This is consistent with previous observations showing that during synaptogenesis, presynaptic differentiation precedes postsynaptic assembly (30). Together, our findings support a model in which STEP functions as a negative regulator of synaptogenesis, acting as a developmental “brake” that constrains the formation and maturation of presynaptic terminals.

Mechanistically, our findings support a model in which STEP acts as a phosphatase checkpoint at developing presynaptic boutons. We show that phosphorylated (inactive) STEP accumulates at presynaptic sites during synaptogenesis, and its genetic or pharmacological inhibition enhances vesicle release and network synchrony. Conversely, elevated STEP activity in *Fmr1* KO neurons reduces presynaptic differentiation and suppresses ERK phosphorylation, a canonical STEP substrate. These results place STEP upstream of signaling pathways that regulate presynaptic assembly and provide a conceptual framework in which local STEP inactivation is permissive for bouton maturation and synaptic function.

To distinguish between presynaptic versus postsynaptic STEP contributions to the process of synapse formation, we established a circuit-on-a-chip system between STEP^−/−^ axons and WT dendrites using microfluidic devices. Our results show that genetic deletion of STEP specifically from presynaptic neurons leads to an increase in the number of axodendritic synapses. Previous studies have shown that protein tyrosine phosphatases, namely the nonreceptor types like STEP, are involved in presynaptic formation through various cellular processes, including cytoskeletal dynamics, vesicle trafficking, and signal transduction (31–33). This leads to an exciting model in which STEP, acting in axonal domains, may locally regulate the formation of presynaptic terminals. Additionally, we observed lower–molecular-weight STEP-immunoreactive species in hippocampal synaptosomes during early development. While STEP61 is the major isoform reported in the hippocampus, STEP isoform patterns are known to vary across brain regions and developmental stages (9, 34–37). As the focus of this study is to define STEP’s developmental role in presynaptic assembly, a detailed characterization of isoform biology lies beyond the scope of the current work, but these observations highlight an intriguing area for future investigation.

Collectively, our findings raise the question of which proteins are targeted by presynaptic STEP. The currently accepted model suggests that STEP hinders synaptic strengthening by dephosphorylating and inactivating key target proteins. These include neurotransmitter receptors and signaling molecules (1–4, 6); however, at the presynaptic side, the information is far scarcer. One of the best-characterized substrates of STEP is ERK1/2, a central regulator of phosphorylation-dependent signaling pathways (2, 17). In developing neurons, ERK signaling contributes to axon growth, branching, and growth cone dynamics, and also operates locally at presynaptic terminals to regulate synaptic vesicle mobilization and neurotransmitter release (38–45). Given that STEP directly dephosphorylates and inactivates ERK1/2, these observations support a model in which STEP limits presynaptic development by restraining ERK-dependent phosphorylation events involved in bouton maturation and vesicle dynamics. Consistent with this idea, we observed increased STEP activity in *Fmr1* KO hippocampal synaptosomes, together with reduced ERK1/2 phosphorylation and impaired presynaptic differentiation. These findings support a role for STEP-dependent phosphatase activity in regulating ERK signaling during presynaptic maturation, although further work will be required to determine whether ERK is the principal mediator of STEP function at the presynapse. In addition, proteomic analyses of the STEP61 interactome identified candidate substrates linked to cytoskeletal regulation, motor function, and vesicle trafficking (46), raising the possibility that STEP may also influence presynaptic development through broader control of proteins required for bouton formation and synaptic vesicle dynamics (47–49). Determining whether ERK1/2 and/or additional cytoskeletal and vesicle-trafficking proteins identified by proteomic studies act as presynaptic substrates of STEP will be important to further define this regulatory pathway.

Notably, functional imaging using an FM1-43 dye-based assay demonstrated that the additional synaptic puncta observed in STEP^−/−^ neurons are capable of activity-dependent vesicle cycling, confirming that they are functionally active rather than immature or nonfunctional (“silent”) synapses. Moreover, while the proportion of moderately active synapses was similar between genotypes, STEP^−/−^ cultures exhibited a significant increase in highly active synapses, indicating elevated presynaptic release capability. These findings further support a model in which STEP functions as a molecular brake that restrains presynaptic differentiation and neurotransmission efficacy during early circuit assembly. Further multielectrode array data revealed that STEP^−/−^ hippocampal cultures exhibited a higher mean firing rate, along with an increased number of bursts and a higher synchrony index, without significant changes in network burst frequency. This phenotype indicates a more mature and coordinated network, consistent with greater presynaptic function and strengthened synaptic connectivity, while global network dynamics remain temporally stable. These findings are in line with previous studies showing that synapse formation is closely associated with the development of synchronized neuronal activity (50, 51). However, despite this local increase in excitability, the overall network rhythm remained stable, suggesting that homeostatic and/or inhibitory mechanisms at the network level preserve the temporal structure of synchronized events. In this sense, we cannot exclude the possibility that the increased neuronal excitability observed in STEP^−/−^ cultures may also reflect a possible decrease in GABAergic transmission. In fact, inhibitory GABAergic input regulates firing behavior, such that a decrease in GABAergic neurotransmission causes the firing pattern to shift to a bursting state (51, 52). Thus, it is important to stress the possibility that STEP can also modulate the formation of inhibitory synapses during neuronal development. Indeed, three distinct studies propose that STEP might serve as a key intermediary for GABAergic transmission (19, 53, 54). Future studies assessing the excitatory/inhibitory balance after modulating STEP function will therefore offer a more comprehensive understanding of the effects of STEP on neuronal network activity during neuronal development.

Increased STEP levels in the FXS mouse model have been associated with several features of this pathology. We observed that STEP levels are increased in the axons of hippocampal neurons from *Fmr1* KO mice. Moreover, increased STEP activity and a reduction in the phosphorylation of its substrate, ERK1/2 (pERK1/2; Thr202/Tyr204), were observed in synaptosomal fractions from *Fmr1* KO mice compared to WT controls. We also found an impairment in presynaptic differentiation in hippocampal neurons from *Fmr1* KO mice, suggesting that presynaptic STEP may underlie these deficits. Notably, genetic or pharmacological manipulation of STEP reverses behavioral, electrophysiological, and dendritic spine abnormalities in *Fmr1* KO mice (14, 15). Interestingly, by rendering STEP inactive in *Fmr1* KO neurons using the TAT-STEP C-S peptide, which acts as a substrate-trapping mutant, we were able to enhance presynaptic terminal assembly to levels comparable to the control, raising the possibility that STEP inhibition could be an effective approach for ameliorating some pathological features of FXS.

Altogether, we show that STEP inhibition enhances presynaptic assembly. We propose that in the developing brain, high STEP activity acts as a brake on the formation of presynaptic sites, and spatiotemporal reduction of this activity leads to increased synaptogenesis. One possible mechanism for this endogenous inhibition may be through Protein Kinase A (PKA), which negatively regulates STEP activity by maintaining it in a phosphorylated inactive state (55–57). In fact, during synaptogenesis, PKA activity is known to increase and plays a critical role in promoting synaptic differentiation (58, 59). It is therefore plausible that increased PKA activity during synaptogenesis leads to STEP phosphorylation and inactivation, thereby relieving its inhibitory effect on downstream signaling pathways, including ERK1/2, and promoting mechanisms that support presynaptic development. Interestingly, reduced PKA activity and expression has been associated with neurodevelopmental disorders like FXS (60–63), further supporting this hypothesis. Consistent with this model, p-STEP was preferentially localized at glutamatergic presynaptic terminals, indicating that local inactivation of STEP at axonal sites may serve as a permissive signal for synapse formation during neuronal development. Taken together, these findings suggest that tightly regulated STEP activity is critical for neurodevelopment, and its dysregulation may contribute to the synaptic deficits observed in neurodevelopmental disorders such as FXS.

## Materials and Methods

Primary hippocampal neurons were isolated from embryonic mouse and rat brains and maintained in neurobasal-based medium under standard serum-free conditions (64). Lentiviral construct expressing fluorescent reporter was produced in HEK293T cells by cotransfection with packaging plasmids and used to transduce neurons at DIV6. Biochemical analysis of synaptosomes was performed from hippocampal tissue lysates separated by differential centrifugation and density gradients, followed by SDS-PAGE and immunoblotting. Immunocytochemistry and immunohistochemistry were carried out using antibodies against synaptic and neuronal markers, with confocal or widefield imaging for quantitative analysis of protein localization and synaptic structures. Microfluidic chambers assay was carry on polydimethylsiloxane (PDMS) chamber assembled on a glass coverslip and were prepared as described previously with some modifications (24). TAT-Myc and TAT-STEP peptides were kindly offered by Dr. Paul J. Lombroso and synthesized by the core facility at Yale University (New Haven, CT) as previously described (29, 65). Synaptosomes were obtained from hippocampal tissue through a discontinuous Percoll gradient as previously described (66). STEP-specific phosphatase activity was determined in hippocampal synaptosomes immunoprecipitated with anti-STEP antibodies using Protein A/G agarose beads, and activity was measured by incubating the immune complexes with the p-nitrophenyl phosphate (pNPP) substrate and reading absorbance at 405 nm. Functional synapses were assessed by measuring synaptic vesicle release using an FM1-43 dye-based assay, and neuronal network activity was recorded with multielectrode arrays (MEAs) using M384-tMEA 24-well plates (Axion Biosystems, Atlanta, GA, USA). For more detailed methodology and information on antibody details, fixation and staining protocols, image acquisition and analysis, and statistical procedures, see SI Materials and Methods.

## Supplementary Material

Appendix 01 (PDF)

## Data Availability

All study data are included in the article and/or *SI Appendix*.
