# Supplementary material for: The tyrosine phosphatase STEP is a developmental suppressor of synaptogenesis
Source: Proc Natl Acad Sci U S A. 2026 Jun 10;123(24):e2424788123. doi: 10.1073/pnas.2424788123 (PMC13273286; doi:10.1073/pnas.2424788123)
Supplement: Supplementary file 1 — Appendix 01 (PDF) [file pnas.2424788123.sapp.pdf]

PNAS

Supporting Information for

The tyrosine phosphatase STEP is a developmental suppressor of synaptogenesis

Joel P. Pires[1,2,3,4,5], Diogo Tomé[1,2,5], Miranda Mele[1,2], Ana Caulino-Rocha[2,6,7], Elisa Corti[1,2,8,9], Ira Milosevic[6,10], Graça F. Baltazar[3,4], Ramiro D. Almeida[1,2,5]*

*Correspondence to Ramiro D. Almeida
Email: ramirodalmeida@gmail.com

This PDF file includes:

Supporting Materials and Methods
References for SI Materials and Methods
Supplementary Figures S1–S3 and corresponding figure legends

SI Materials and Methods

Primary Neuronal Cultures

Rat embryonic hippocampal neurons

Primary cultures of rat embryonic hippocampal neurons were prepared from the hippocampi of embryonic day 17–18 Wistar rat embryos as described previously (1). Briefly, after dissection, hippocampi were dissociated in 0.045% trypsin and 0.01% v/v deoxyribonuclease in Hank's balanced salt solution (HBSS) [5.36 mM KCl, 0.44 mM KH_2PO_4, 137 mM NaCl, 4.16 mM $NaHCO_3$, 0.34 mM $Na_2HPO_4 \cdot 2H_2O$, 5 mM glucose, 1 mM sodium pyruvate, 10 mM N-(2-hydroxyethyl)piperazine-N'-(2-ethanesulfonic acid) (HEPES), and 0.001% phenol red] for 15 minutes at 37°C. Hippocampi were then centrifuged at 200 × g for 1 minute at room temperature (RT) and washed once in plating medium (MEM supplemented with 10% FBS, 0.6% glucose, and 1 mM pyruvic acid) with 0.01% v/v deoxyribonuclease and centrifuged again at 200 × g for 1 minute at RT. The pellet was mechanically dissociated in fresh plating medium, and cell density was determined. Cells were plated in plating medium on PDL-coated surfaces as follows: to create pseudoexplants for the isolation of axons, 2.5×10^4 cells were plated in plating medium on poly-D-lysine-coated glass coverslips inside a cylinder (6 mm diameter) placed in the center of a coverslip-containing well of a 24-well plate; for multielectrode array recordings, 10 µL of cell suspension containing 1×10^4 cells and 10 µg/mL laminin were plated in the inner circle of the well. Neurons were allowed to attach for 2-4 hours, and then the plating medium was replaced with culture medium (Neurobasal medium supplemented with 2% B27, 25 µM glutamate, 0.5 mM glutamine, and 1:400 penicillin-streptomycin). Cells were maintained in a humidified incubator with 5% CO_2/95% air at 37°C. At DIV3, the mitotic inhibitor 5-fluoro-2′-deoxyuridine (5-FDU) (10 µM final concentration) was added to avoid glia overgrowth. Cells were allowed to grow, and unless otherwise indicated, experiments were performed at DIV9 or DIV12.

Mouse embryonic hippocampal neurons

Primary cultures of mouse hippocampal neurons were prepared from the hippocampi of E15.5 $STEP^{-/-}$ (B6N.129-Ptpn5^{tm1Pijlo}/J) and wild-type mouse embryos on a C57BL/6J background (2). Hippocampi were dissected and dissociated with papain (20 U/mL) and 0.01% v/v deoxyribonuclease in Hank's balanced salt solution (HBSS) [5.36 mM KCl, 0.44 mM KH_2PO_4, 137 mM NaCl, 4.16 mM $NaHCO_3$, 0.34 mM $Na_2HPO_4 \cdot 2H_2O$, 5 mM glucose, 1 mM sodium pyruvate, 10 mM N-(2-hydroxyethyl)piperazine-N'-(2-ethanesulfonic acid) (HEPES), and 0.001% phenol red] with $CaCl_2$ (1.5 mM), EDTA (0.5 mM), and L-cysteine (1.65 mM) for 10 minutes at 37°C. Hippocampi were then centrifuged at 200 × g for 1 minute at room temperature (RT) and washed once in plating medium (MEM supplemented with 10% horse serum, 0.6% glucose, 1 mM pyruvic acid, and 4.3 mM $NaHCO_3$) with BSA (0.25% w/v) and trypsin inhibitor (0.25% w/v) to stop papain activity. Three additional washes were done in pre-warmed HBSS, and the pellet was mechanically dissociated in fresh plating medium, and cell density was determined. Cells were plated in plating medium on PDL-coated surfaces as follows: to create pseudoexplants for the isolation of axons, 2.5×10^4 cells were plated in plating medium on poly-D-lysine-coated glass coverslips inside a cylinder (6 mm diameter) placed in the center of a coverslip-containing well of a 24-well plate; for synapse formation analysis using microfluidic devices, 8×10^4 cells of WT and/or $STEP^{-/-}$ genotypes were plated in the presynaptic and postsynaptic compartments (see Microfluidic chambers assay method). Neurons were allowed to attach for 2-4 hours, and then the plating medium was replaced with culture medium (Neurobasal medium supplemented with 2% B27, 25 µM glutamate, 0.5 mM glutamine, and 1:400 penicillin-streptomycin). Cells were maintained in a humidified incubator with 5% CO_2/95% air at 37°C. At DIV3, the mitotic inhibitor 5-fluoro-2′-deoxyuridine (5-FDU) (10 µM final concentration) was added to avoid glia overgrowth. Cells were allowed to grow, and unless otherwise indicated, experiments were performed at 9 or DIV12.

Mouse postnatal hippocampal neurons

Primary cultures of hippocampal neurons from *Fmr1* KO mice and their WT littermates were prepared from postnatal day 0 (P0) hippocampi of B6.129P2-Fmr1^{tm1Cgr}/J mice (The Jackson Laboratory) after genotyping by tail-PCR using the hot sodium hydroxide and tris (HotSHOT)

protocol (3). After dissection, hippocampi were dissociated in 0.05% trypsin in Hank's balanced salt solution (HBSS) [5.36 mM KCl, 0.44 mM KH_2PO_4, 137 mM NaCl, 4.16 mM $NaHCO_3$, 0.34 mM $Na_2HPO_4 \cdot 2H_2O$, 5 mM glucose, 1 mM sodium pyruvate, 10 mM N-(2-hydroxyethyl)-piperazine-N'-(2-ethanesulfonic acid) (HEPES), and 0.001% phenol red] for 8 minutes at 37°C. After enzymatic digestion, HBSS with 10% FBS was added to block trypsin action, followed by centrifugation at 200 × g for 5 minutes at room temperature (RT). The tissue pellets were mechanically dissociated in fresh plating medium and cell density determined. To create pseudoexplants for the isolation of axons, 2.5 × 10^4 cells were plated in Neurobasal medium A (NBM A) supplemented with 2% B27, 0.5 mM glutamine, and 10 μg/mL gentamicin onto poly-D-lysine-coated glass coverslips inside a cylinder (6 mm diameter) placed in the center of a coverslip-containing well of a 24-well plate. Neurons were allowed to attach for 1-2 hours, after which the cylinder was removed, and the supplemented NBM A was replaced with fresh pre-wormed supplemented NBM A. Cells were maintained in a humidified incubator with 5% CO_2/95% air at 37°C. At DIV3, the mitotic inhibitor 5-fluoro-2′-deoxyuridine (5-FDU) (10 μM final concentration) was added to avoid glia overgrowth. Cells were allowed to grow, and unless otherwise indicated, experiments were performed at DIV9 or DIV12.

<u>Low-density hippocampal cultures</u>
Low-density hippocampal cultures were prepared from E15.5 wild-type and $STEP^{-/-}$ (B6N.129-$Ptpn5^{tm1Pijlo}$/J) mice embryos on a C57BL/6J background by plating the dissociated cells at a final density of 2.5 × 10^5 cells per dish (60 mm ×15 mm), each containing five poly-D-lysine-coated glass coverslips (18 mm) in neuronal plating medium (MEM supplemented with 10% FBS, 0.6% glucose, and 1 mM pyruvic acid). After 2 - 4 hours, coverslips were flipped over an astroglial feeder layer in Neurobasal medium supplemented with 2% B27, 25 μM glutamate, 0.5 mM glutamine, and 1:400 penicillin-streptomycin. The neurons grew face down over the feeder layer but were kept separate from the glia by paraffin dots on the neuronal side of the coverslips. To prevent overgrowth of glial cells, neuron cultures were treated with the mitotic inhibitor 5-fluoro-2′-deoxyuridine (5-FDU) (10 μM final concentration) at DIV3. Cultures were maintained in a humidified incubator with 5% CO_2/95% air at 37°C. Cells were allowed to grow, and experiments were performed at 9 or DIV15. Astroglial feeder layers were prepared from rat embryos, with 20,000 to 40,000 cells plated per 60 mm dish and cultured in Minimum Essential Medium containing 0.6% glucose, 1% penicillin-streptomycin, and 10% heat-inactivated horse serum for 14 days.

Microfluidic chambers assay
Microfluidic devices consist of a molded polydimethylsiloxane (PDMS) chamber assembled on a glass coverslip and were prepared as described previously with some modifications (4). Briefly, PDMS devices were prepared using Sylgard 184 Silicone Elastomer Kit (Dow Corning). The pre-polymer mixture was added to a master mold fabricated and kindly provided by Noo Li Jeon (School of Mechanical & Aerospace Engineering, Seoul National University, Seoul 151-472, Korea), placed in a vacuum chamber to remove any air bubbles for 30 minutes, and cured for 4-6 hours at 60°C. Before being assembled on a glass coverslip, PDMS chambers were properly cleaned, sterilized with 70% ethanol, and air-dried in the culture hood. Glass coverslips (Marienfeld #0101222) were cleaned in 65% nitric acid for 24 hours, washed 5 times (30 minutes each wash) with mQH_2O, rinsed twice in 100% ethanol, dried, and sterilized under UV radiation for at least 15 minutes. The PDMS mold was properly assembled on the glass coverslip under sterile conditions. Prior to plating cells all reservoirs of each PDMS device, previously assembled on a PDL-coated glass coverslip were filled with plain Neurobasal medium containing laminin (2 μg/mL) and incubated for 2 hours at 37°C. The PDMS devices were then washed once with plain Neurobasal medium without laminin and once with plating medium (MEM supplemented with 10% horse serum, 0.6% glucose, 1 mM pyruvic acid, and 4.3 mM $NaHCO_3$). For conventional two-compartment chambers, 5 × 10^4 neurons were seeded in the somal compartment, and their axons were allowed to extend through the microgrooves into the axonal compartment. For synapse formation chambers, 8 × 10^4 cells of the WT genotype were plated in each presynaptic and postsynaptic compartment ("WT chamber"), and 8 × 10^4 cells from $STEP^{-/-}$ and WT genotypes were plated in the presynaptic and postsynaptic compartments, respectively ("$STEP^{-/-}$

chamber"). At DIV6, 2/3 of the conditioned media were collected from the presynaptic reservoirs and stored at 37°C. Viral particles of VGluT1-mCherry were added to the presynaptic compartment, and the microfluidic devices were incubated for 6 hours at 37°C. After 6 hours, the presynaptic compartments were washed twice with Neurobasal medium, and the previously removed conditioned media were added back to the respective compartment. From DIV6 to DIV9, a volume difference was maintained between the two compartments (more in the postsynaptic compartment) to create high fluidic resistance and ensure fluidic isolation of the viral particles in the presynaptic compartment. At DIV9, cells were fixed, and immunocytochemistry was performed.

Generation of Lentivirus

For the generation of lentivirus, HEK293T cells were transfected with the lentiviral expression vector [F(syn)WRBN-VGluT1-mCherry] and the lentiviral packaging vectors: psPAX2 and pLP/VSVG using PEI (Polysciences) at 1:6 DNA/PEI ratio. Six hours after transfection, the medium was changed to DMEM (Sigma) without FBS and antibiotics. Conditioned media containing viral particles were collected 72 hours after transfection and centrifuged at 250 × g for 10 minutes at 4°C. The supernatant was sterile-filtered using a 0.45 μm cellulose acetate membrane filter and concentrated by centrifugation at 80,000 × g for 2 hours at 22°C, using a Beckman XE-100 ultracentrifuge with an SW41 Ti rotor. The viral pellet was then resuspended in PBS with 0.1% BSA and stored at -80°C.

Generation and purification of TAT-myc and TAT-STEP peptides

The TAT-STEP peptides were kindly offered by Dr. Paul J. Lombroso and synthesized by the core facility at Yale University (New Haven, CT) as previously described (5, 6). Three different peptides were used: the TAT-STEP46 C-S (2 μM), a TAT-based membrane-permeable peptide, which contains a point mutation in its catalytic domain (C300S), rendering an enzymatically inactive STEP and acts as a substrate-trapping mutant; the TAT- STEP46 WT (2 μM), a constitutively active form of STEP; and an inactive TAT-myc peptide (2 μM) that was used as a control. For all experiments with TAT peptides, cells were exposed to the peptides for 1 h. These STEP fusion peptides exhibited highly efficient cellular uptake, resulting in near-complete transduction across the cell population, and remained detectable within primary neuronal cultures up to 6 hours post-treatment (5, 7, 8). Efficient intracellular delivery of TAT-peptides was confirmed by immunocytochemistry against the Myc-Tag in DIV9 hippocampal neurons following 1 h incubation.

Synaptosome preparation

Synaptosomes were obtained from hippocampal tissue through a discontinuous Percoll gradient as previously described (9). Briefly, hippocampal tissue was homogenized in ice-cold isolation sucrose solution (0.25 M sucrose, 10 mM HEPES, pH 7.4 at 4°C) using a glass-Teflon tissue grinder. Nuclei and debris were removed by centrifugation (3,000 × g, 5 min at 4°C), and the supernatant was centrifuged at 13,000 × g for 10 min at 4°C. The pellet was resuspended in 2 mL of ice-cold 0.25 M sucrose and carefully placed on top of a discontinuous gradient composed of 23%, 10%, 6%, and 2% v/v Percoll in sucrose solution (0.32 M sucrose, 1 mM EDTA, pH 7.4 at 4°C), which was stratified by centrifugation (31,000 × g for 5 min at 4°C), turning off the centrifuge brake for the last 2,000 × g to avoid a sudden stop. Synaptosomes were carefully collected from the interface between the layers containing 10% and 23% Percoll. For each 1 mL of synaptosomes, 10 mL of isotonic physiological solution (115 mM NaCl, 3 mM KCl, 1.2 mM KH_2PO_4, 2 mM HEPES, 10 mM glucose, 1.2 mM $MgSO_4$, 1 mM $CaCl_2$) was added, and the preparation was centrifuged at 30,000 × g for 20 min at 4°C. The pellet containing synaptosomes was washed by adding 1.5 mL of isotonic solution and centrifuged for 20 min at 22,000 × g at 4°C. The resulting pellet was gently resuspended in 400 μL of isotonic solution, and the preparation was used for immunocytochemistry and biochemical analysis.

Biochemistry

Biochemical analysis of synaptosomes was performed by Western blot. After synaptosome preparation, fraction 4 (F4), containing purified synaptosomes (or the other samples used for

blotting) was sonicated for 3 cycles of 10 seconds each at 40 Hz, centrifuged at 16,100 × g for 10 minutes at 4°C, and the supernatant was collected. Quantification of protein was performed using the BCA assay, and samples (40 µg in 40 µL) were denatured with denaturing buffer and boiled at 95°C for 5 minutes. Protein extracts were electrophoresed in a tris-glycine-SDS buffer (25 mM Tris, 192 mM glycine, 0.1% w/v SDS, pH 8.3) in a 10% polyacrylamide gel, 1.5 mm thick. Electrotransfer onto a nitrocellulose membrane was performed at 200 mA for 2 hours at 4°C. Membranes were washed once with TBS (20 mM Tris, 137 mM NaCl) with 0.1% v/v Tween 20 (TBS-T) and once with TBS. Then, membranes were blocked in TBS-T with 5% BSA for 1h or 5% non-fat dry milk for 2h at room temperature (RT). Membranes were washed again twice with TBS-T and incubated overnight at 4°C with the primary antibody diluted in TBS-T containing 1% non-fat dry milk or 1% BSA. The antibodies used were anti-STEP (clone 23E5, 1:200; #4396S, Cell Signalling), anti-Synaptophysin (clone YE269, 1:200000; ab32127, Abcam), anti-ERK1/2 (clone W15133B, 1:1000; #686901, BioLegend), anti-p-ERK Tyr204/187 (clone 6B8B69, 1:1000; #369501, BioLegend), anti-β-Actin (clone AC-74, 1:2000; #A2228, Sigma) and anti-alpha-tubulin (clone 11H10, 1:1000; #2125, Cell Signalling). After three washes with TBS-T, membranes were incubated for 1 hour with horseradish peroxidase-conjugated secondary antibodies (anti-mouse, 1:5000, #7074P2, Cell Signalling; anti-rabbit, 1:5000, #7074P2, Cell Signalling) at RT, washed again three times, and developed with an electrochemiluminescence (ECL) substrate (#GERPN2235; Sigma-Aldrich) for 5 minutes. Membranes were scanned with the ChemiDoc™ MP Imaging System (Bio-Rad). Whenever necessary, membranes were stripped with 0.2 M NaOH for 20 minutes and reprobed.

STEP phosphatase assay

To assess STEP-specific phosphatase activity, crude synaptosomes obtained from postnatal day 9 (P9) hippocampal tissue were solubilized in RIPA buffer containing (in mM): 50 Tris-HCl (pH 7.5), 150 NaCl, 5 EGTA, 1% Triton X-100, 0.5% sodium deoxycholate, 0.1% SDS, and protease and phosphatase inhibitors (ThermoFisher). Samples were incubated on ice for 30 minutes and centrifuged at 13,000 × g for 15 minutes at 4 °C before protein quantification by BCA method. The supernatant was precleared using 30µL of 50% (w/v) Protein A/G PLUS agarose beads (Invitrogen) for 2 hours at 4 °C with gentle rotation. Following preclearing, samples were incubated overnight at 4 °C with 2 µg monoclonal anti-STEP or control IgG antibody under continuous rotation. Immune complexes were captured with fresh Protein A/G agarose beads for 2 hours at 4 °C. Beads were first washed 3 times with cold lysis buffer containing phosphates and protease inhibitors, and then with assay buffer (25 mM HEPES, pH 7.0; 20 mM $MgCl_2$) without phosphatase inhibitors. STEP activity was assessed by incubating immunoprecipitated complexes in assay buffer containing 15 mM p-nitrophenyl phosphate (pNPP; Sigma, P7998) at 30 °C for 30 minutes under gentle shaking. The reaction was stopped by the addition of 1 M NaOH. After brief centrifugation, supernatants were transferred to a clean 96-well plate, and absorbance was measured at 405 nm using a microplate reader (BioTek Synergy). Control reactions included IgG immunoprecipitates and samples incubated with phosphatase inhibitors.

Immunocytochemistry

Cells or synaptosomes were fixed in pre-warmed 4% paraformaldehyde (in PBS with 4% sucrose) for 10 minutes at room temperature (RT). Preparations were washed three times in PBS, then permeabilized in PBS with 0.25% Triton X-100 for 5 minutes at RT and washed once in PBS before blocking for 40 minutes in PBS with 3% BSA. The preparations were then incubated with the primary antibodies in 3% BSA overnight at 4°C, washed three times in PBS, and incubated with the secondary antibodies for 1 hour at RT in 3% BSA. Preparations were again washed twice in PBS with 0.1% Triton X-100 and once in PBS and mounted in ProLong mounting medium with or without DAPI. For microfluidic chambers, the PDMS device was disassembled from the coverslip only before mounting on the microscope glass. The following primary antibodies were used: chicken polyclonal anti-MAP2 (1:10,000; #ab5392; Abcam), mouse monoclonal anti-MAP2 (1:500; #M9942; Sigma), chicken polyclonal anti-Neurofilament M (1:1,000; #ab5735; Millipore), guinea pig polyclonal anti-VGluT1 (1:15,000; #ab5905; Millipore), rabbit polyclonal anti-Synapsin I (1:4,000; #ab1543; Millipore), mouse monoclonal anti-Bassoon (1:800; #SAP7F407; Enzo Life Sciences), rabbit monoclonal anti-PSD95 (1:600; #34505; Cell

Signaling), mouse monoclonal anti-PSD95 (clone 6G6-1C9) (1:600; #MA1-045; Thermo Fisher Scientific), mouse monoclonal anti-STEP (23E5) (1:200; #4396S; Cell Signaling), rabbit polyclonal anti-phospho STEP (Ser221/Ser49) (1:200; #AB2208; Millipore), rabbit anti-RFP/mCherry (1:800; # 600-401-379; Rockland) and rabbit monoclonal anti-Myc-Tag (71D10) (1:500; #2278S; Cell Signaling). Appropriate Alexa Fluor-conjugated secondary antibodies 488, 568, and 647 (1:1000, Thermo Fisher Scientific) and AMCA (1:600; Jackson ImmunoResearch) were used.

Immunohistochemistry

$STEP^{-/-}$ and WT mice were anesthetized by inhalation of isoflurane followed and transcranial perfused with ice-cold PBS followed by 4% PFA in PBS. Brains were removed, post-fixed for 24 h in PFA, dehydrated in 30% sucrose solution for 72 h at 4°C and cryopreserved at −80°C. Coronal brain sections (10 µm) were obtained using a cryostat (CryoStar NX50, ThermoScientific, RRIDD:SCR_022732) collected onto microscope slides for further immunostaining. 6 cryostat sections per animal were used for analysis with at least 80 µm distance between them. For immunohistochemistry, coronal sections were washed 3 times in PBS 5 min each and incubated with blocking and permeabilization solution (10% goat serum with 0.3% triton in PBS) for 1h at RT, followed by incubation with primary antibodies overnight at 4ºC (5% goat serum with 0.3% Triton in PBS). Primary antibodies used were as follows: guinea pig polyclonal anti-Homer 1b/c (1:600; #160-023; Synaptic Systems), mouse monoclonal anti-VGluT1 (1:500; #MAB5502; Millipore) and chicken polyclonal anti-MAP2 (1:250; # ab5392; Abcam). Sections were washed 3 times in 0.3% triton in PBS for 15 min each and incubated with secondary antibodies for 3h at RT (5% goat serum with 0.3% triton in PBS). Alexa-conjugated secondary antibodies 488, 568 and 647 (1:1000, Thermo Fisher Scientific) were used. After incubation with the secondary antibody, the sections were washed four times in 0.3% Triton X-100 in PBS for 15 minutes each, then incubated for 20 minutes with Hoechst 33342 (1:1000; #B2261; Sigma-Aldrich). Afterward, the sections were washed three times in PBS for 5 minutes each, and the slides were coverslipped using Fluoroshield™ (#F6182; Sigma-Aldrich).

FM1-43 dye assay of evoked synaptic activity

To assess synaptic functionality, FM1-43 dye uptake and release assay were performed in wild-type and $STEP^{-/-}$ hippocampal neuronal cultures, transduced with the F(syn)WRBN-VGluT1-mCherry plasmid to label excitatory presynaptic boutons. This assay allows to evaluate whether VGluT1-mCherry puncta correspond to active synaptic terminals capable of neurotransmitter release (10). Before imaging, cultured neurons on coverslips were transferred to wells containing a saline solution (in mM): 125 NaCl, 2 KCl, 2 $CaCl_2$, 2 $MgCl_2$, 30 glucose, 25 HEPES, adjusted to 310 mOsm, pH 7.4. To load synaptic vesicles with FM1-43, cells were incubated for 2 min at room temperature in a high-potassium staining solution (in mM): 82 NaCl, 45 KCl, 2 $CaCl_2$, 2 $MgCl_2$, 30 glucose, 25 HEPES, 310 mOsm, pH 7.4. This depolarizing solution promoted vesicle fusion and dye internalization. Following staining, neurons were washed with the initial saline solution supplemented with CNQX (10 µM), AP5 (50 µM), and tetrodotoxin (1 µM) to suppress spontaneous activity. A final wash with saline containing CNQX and AP5 was performed for 5 min at room temperature to remove residual dye from the plasma membrane and extracellular space. To evaluate FM1-43 dye release, we examined destaining induced by a stimulating solution containing 50 mM KCl, dissolved in the previously described saline solutions and supplemented with CNQX and AP5. This high-potassium solution induces synaptic vesicle release via direct membrane depolarization. For fluorescent imaging, the preparation was excited at 488 nm using a solid-state 100 mW laser, and images were acquired using a Zeiss Cell Observer Spinning Disk microscope. The system was equipped with a Plan-Apochromat 63×, 1.3 NA objective lens and an Electron Multiplying-CCD Evolve Delta (Teledyne Photometrics) camera. This configuration allowed for high-quality imaging of synaptic activity during the experiment. A high-magnification (63x) objective lens was used to confirm the health of the neurons in the imaging field under transmitted light microscopy. The FM dye fluorescence was recorded at 1-second intervals, and the images were analyzed using ImageJ software for quantification of vesicle release events. The intensity of fluorescence was used to assess the presence and dynamics of synaptic vesicle release, and the kinetics of dye release were used to infer the functional status of the synaptic

terminals during evoked activities. The change in FM1-43 fluorescence intensity (ΔF) was used to assess synaptic activity following depolarization. Images were analyzed before (t_0) and 60 seconds after stimulation (t_f). For each synaptic punctum, the fluorescence intensity was measured at both time points, and ΔF was calculated as the difference between the initial (mean of 3 points) and final fluorescence values. This ΔF value represents the amount of FM1-43 dye released from presynaptic terminals during stimulation, reflecting synaptic vesicle exocytosis and, therefore, synaptic activity. Synapses were categorized based on the extent of fluorescence loss into two groups: Moderately functional synapses: ($5 < \Delta F < 20$) and Highly active synapses: ($\Delta F > 20$). The number of active synapses per unit dendritic length (µm) was quantified for both WT and $STEP^{-/-}$ neurons.

Multielectrode array recordings and data analysis

Multielectrode array recordings were obtained from mice embryonic hippocampal neuronal cultures (10K cells/well; prepared as described previously) plated in M384-tMEA-24 well plates (Axion Biosystems, Atlanta, GA, USA). At DIV3, the mitotic inhibitor 5-fluoro-2′-deoxyuridine (5-FDU) (10 µM final concentration) was added to reduce contamination with glia cells. At DIV7, 150 µL of conditioned media was taken out of each well, and 150 µL of fresh Neurobasal medium supplemented with 2% B27, 0.5 mM glutamine, and 1:400 penicillin-streptomycin was added. At DIV14, simultaneous recordings from 16 extracellular electrodes per well were obtained using the Maestro microelectrode array system (Axion Biosystems) at a constant temperature of 37°C and in an atmosphere of 5% CO2/95% air, for 10 min. Data were sampled at 12.5 kHz and digitized and analyzed using Axion Integrated Studio software (Axion Biosystems). Spike events were detected using an adaptive spike detection threshold of 5.5 SD of noise for each electrode. Single-electrode bursts were defined as at least 5 consecutive spikes with inter-spike intervals shorter than 100 ms. Network bursts were identified using the envelop algorithm with a threshold factor of 1.25, a minimum inter-burst interval of 100 ms, at least 65% of burst inclusion and having a minimum of 70% of electrodes simultaneously active. The recording medium was Neurobasal-conditioned media.

Image acquisition and analysis

Imaging of cultured neurons was performed using a Plan-Apochromat 20x air objective (0.8 numerical aperture) or a Plan-Apochromat 63x oil objective (1.4 numerical aperture) in a Carl Zeiss Observer Z1 microscope equipped with an AxioCam HRm camera and the Zen Blue 2011 software (ZEISS). All experimental conditions within independent preparations were imaged using identical settings (exposure time and fluorescence light intensity were kept constant throughout acquisition). Independent experiments correspond to independent neuronal culture preparations obtained from separate dissections performed on different days using independent animal litters. For imaging-based analyses, multiple random fields of view (FOVs) were acquired from each preparation, with multiple axons or dendrites analyzed per FOV. Biological replication was therefore defined by the number of independent culture preparations, whereas FOVs represented within-preparation sampling units. Images containing isolated axons or dendrites were converted to 8-bit format for quantification. Image of synaptosomes, were captured using a Carl Zeiss Axio Imager Z2 microscope with a Plan-Apochromat 100× oil objective (1.4 numerical aperture), equipped with a Zeiss HRm AxioCam. Fluorescent images of the stratum oriens layer from hippocampal slices were obtained using a Plan-Apochromat 40x oil objective (1.4 numerical aperture) on a Carl Zeiss LSM 710 confocal microscope equipped with a QUASAR detection unit and Zen Black 2012 software (ZEISS). For all experimental conditions, images of the stratum oriens layer of the hippocampal CA1 region were acquired using the same settings (gain and laser power were kept constant in each independent experiment). A fluorescent image of coronal hippocampal slice was obtained with a Plan-Apochromat 20x objective (0.8 numerical aperture) on a Carl Zeiss on a Axio Scan Z.1 microscope. To quantify the presynaptic puncta/clusters along the axons (Fig. 2, 7 and 8), the axonal marker image was used to randomly select axon populations. Axonal length was measured by analyzing a skeletonized version of the axonal marker using the “Analyze Skeleton” plugin in Fiji. The sum of all axonal branches identified in each field of view (FOV) was measured and used as the total axonal length. Bundled axons were excluded from analysis. To quantify the proteins of interest, corresponding images of presynaptic

markers were subjected to a user-defined intensity threshold, in order to have defined protein clusters, and a user-defined background intensity was subtracted to all images. Both settings were maintained constant in all conditions. The “Analyze Particles” function in Fiji software was used to determine the regions of interests (ROIs) of each punctum and protein clusters present in the ROI were analyzed for the number, intensity and area. The total number, integrated density, and area were normalized to the total axonal length in each FOV. For the analysis of VGluT1/Bassoon clusters, the presence or absence of Bassoon signal within VGluT1 ROIs was determined using the 'Combine and Measure' function in Fiji. The total number of VGluT1 puncta count, along with their corresponding integrated density and area that colocalize with Bassoon signal, was normalized by dividing the mean of each parameter by the total axonal length. To quantify axodendritic synapses (VGluT1/PSD-95 clusters) in low-density hippocampal cultures (Fig. 3), dendrites of similar thickness and appearance were randomly selected based on the MAP2 signal, and the sum of all dendritic lengths identified in each neuron was used as the total dendritic length. In tripartite microfluidic chambers (Fig. 4), images of random FOVs were acquired from the synaptic compartment based on the MAP2 signal, and the axodendritic synapses were quantified based on the VGluT1-mCherry/PSD-95 clusters. To identify and measure individual VGluT1-mCherry puncta and the VGluT1-mCherry/PSD-95 clusters, the workflow described above was used. The final values obtained from the analysis of low-density hippocampal cultures were normalized to the total dendritic length, while the values from microfluidic chamber images were normalized to the total dendritic length in the region where transduced axons overlapped. The puncta/cluster and colocalization analysis in hippocampal slices at stratum oriens layer (Fig. 3), were conducted on 7 confocal z-planes (z resolution of 0.5 μm) with 3× zoom factor and 8 times pixel averaging, after Sum slices projection using the “Z Projection” plugin in Fiji. All analyses were carried out on ICY software (icy.bioimageanalysis.org) (11) using the “Spot Detector” plugin, and colocalization of pre- and postsynaptic clusters analysis was conducted based on the statistical method SODA (Statistical Object Distance Analysis) (12), within the same software. Three images per section from three cryostat sections of each brain, from at least five animals per group, were analyzed.
All images were processed and prepared for presentation using ImageJ/Fiji and Illustrator (Adobe) software.

Statistical analysis
Results are presented as averaged values ± standard error of the mean (SEM). Graphs and statistical analysis were performed in Graph Pad Prism 8.0.1 software. The normality of population distributions was calculated for each experiment by comparison with a theoretical distribution using the Shapiro-Wilk or Kolmogorov-Smirnov normality test. According to this evaluation parametric or non-parametric tests were used, as described in the figure legends.

References for SI Materials and Methods

1. M. J. Pinto, J. R. Pedro, R. O. Costa, R. D. Almeida, Visualizing K48 ubiquitination during presynaptic formation by ubiquitination-induced fluorescence complementation (UiFC). *Front. Molec. Neurosci.* **9** (2016).

2. D. V. Venkitaramani, *et al.*, Knockout of STriatal enriched protein tyrosine phosphatase in mice results in increased ERK1/2 phosphorylation. *Synapse* **63**, 69–81 (2009).

3. G. E. Truett, *et al.*, Preparation of PCR-Quality Mouse Genomic DNA with Hot Sodium Hydroxide and Tris (HotSHOT). *BioTechniques* **29**, 52–54 (2000).

4. M. J. Pinto, *et al.*, The proteasome controls presynaptic differentiation through modulation of an on-site pool of polyubiquitinated conjugates. *J. Cell Biol.* **212**, 789–801 (2016).

5. S. Paul, *et al.*, The Striatal-Enriched Protein Tyrosine Phosphatase Gates Long-Term Potentiation and Fear Memory in the Lateral Amygdala. *Biol. Psychiatry* **61**, 1049–1061 (2007).

6. R. Poddar, I. Deb, S. Mukherjee, S. Paul, NR2B-NMDA receptor mediated modulation of the tyrosine phosphatase STEP regulates glutamates induced neuronal cell death. *J. Neurochem.* **115**, 1350–1362 (2010).

7. R. Tashev, *et al.*, A Substrate Trapping Mutant Form of Striatal-Enriched Protein Tyrosine Phosphatase Prevents Amphetamine-Induced Stereotypies and Long-Term Potentiation in the Striatum. *Biol. Psychiatry* **65**, 637–645 (2009).

8. I. Deb, *et al.*, Neuroprotective Role of a Brain-Enriched Tyrosine Phosphatase, STEP, in Focal Cerebral Ischemia. *J. Neurosci.* **33**, 17814–17826 (2013).

9. P. R. Dunkley, P. E. Jarvie, P. J. Robinson, A rapid percoll gradient procedure for preparation of synaptosomes. *Nat. Protoc.* **3**, 1718–1728 (2008).

10. M. J. Pinto, R. D. Almeida, Puzzling out presynaptic differentiation. *J. Neurochem.* **139**, 921–942 (2016).

11. F. De Chaumont, *et al.*, Icy: An open bioimage informatics platform for extended reproducible research. *Nat. Methods* **9**, 690–696 (2012).

12. T. Lagache, *et al.*, Mapping molecular assemblies with fluorescence microscopy and object-based spatial statistics. *Nat. Commun.* **9** (2018).

Supplementary Figure and Figure Legends

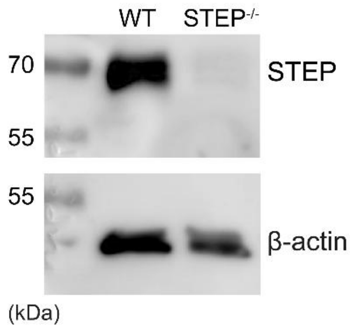

Fig. S1. Validation of anti-STEP antibody specificity using STEP knockout tissue.

Western blot showing anti-STEP antibody specificity in brain homogenates from WT and STEP⁻/⁻ adult mice (B6N.129-Ptpn5tm1Pijlo/J) on a C57BL/6J background. STEP immunoreactivity is detected in WT samples but absent in STEP⁻/⁻ samples, confirming antibody specificity. β-actin was used as a loading control.

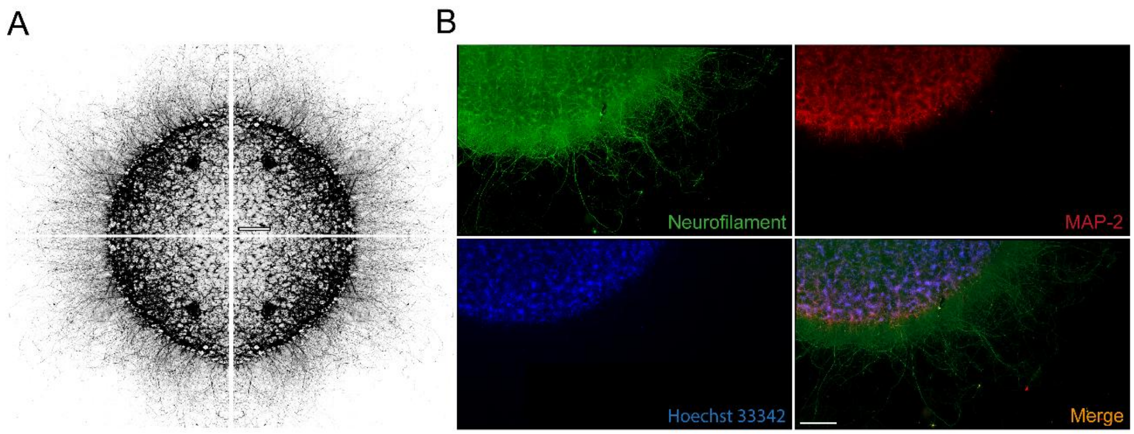

Fig. S2. Pseudoexplants of hippocampal neurons at the early stage of neuronal development.

(*A*) Schematic representation of a pseudoexplant. Binary composition of an image taken with an inverted microscope Zeiss Axiovert Observed Z1, equipped with an AxioCamHRm camera and a plan-Apochromat 20x objective. Scale bar, 500 μm.

(*B*) Widefield fluorescent images of hippocampal pseudoexplant. Hippocampal neurons were immunostained at DIV7 against the axonal marker neurofilament (green) and the somatodendritic marker MAP-2 (red) and nuclei were stained with Hoechst 33342 (blue). Isolated axons growth outwards from the explant and are used for presynaptic puncta analysis. Scale bar, 500 μm.

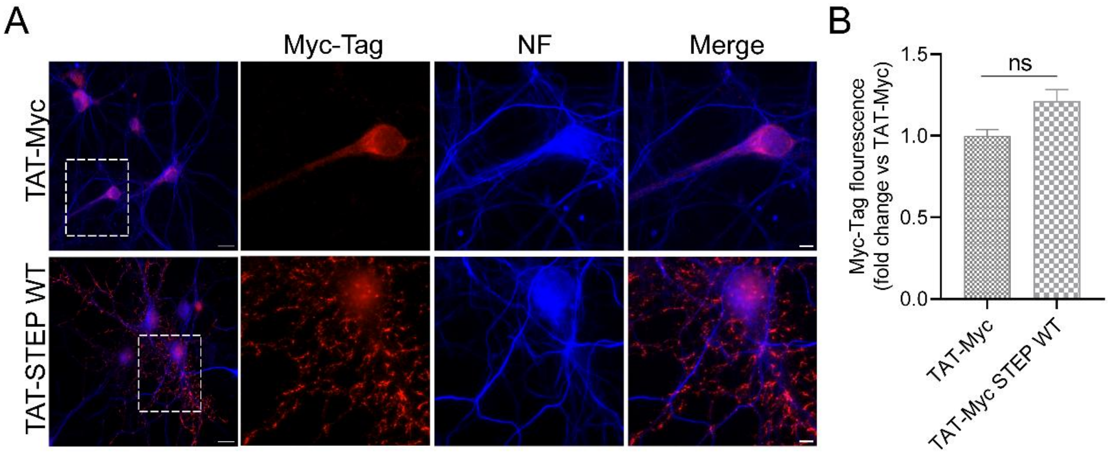

Fig. S3. Validation of neuronal uptake and intracellular levels of TAT-Myc and TAT-Myc-STEP WT.

(*A*) Representative immunocytochemistry images of DIV9 hippocampal neurons incubated for 1 h with TAT-Myc control peptide or TAT-Myc-STEP WT and stained for the Myc epitope tag (red) and neurofilament (NF, blue). Dashed boxes indicate regions shown at higher magnification. Scale bars: 20 µm (low-magnification images) and 5 µm (higher-magnification insets).

(B) Quantification of somatic Myc fluorescence (right) revealed comparable intracellular levels between TAT-Myc and TAT-Myc-STEP WT. Data are expressed as fold change relative to TAT-Myc. Bars represent mean ± SEM of 50–64 cells from 3 independent experiments. Statistical significance was assessed by the non-parametric Mann-Whitney test; ns = not significant.
